# SHEA/APIC/IDSA/PIDS multisociety position paper: Raising the bar: necessary resources and structure for effective healthcare facility infection prevention and control programs

**DOI:** 10.1017/ice.2025.73

**Published:** 2025-07

**Authors:** Thomas R. Talbot, Christopher Baliga, Rebecca Crapanzano-Sigafoos, Tania N. Bubb, Mohamad Fakih, Thomas G. Fraser, Ibukunoluwa C. Kalu, Vidya Mony, Anupama Neelakanta, Ann-Christine Nyquist, Catherine O’Neal, Jan E. Patterson, David K. Warren, Sharon B. Wright

**Affiliations:** 1 Vanderbilt University Medical Center, Nashville, TN, USA; 2 Virginia Mason Hospital, Seattle, WA, USA; 3 The Association for Professionals in Infection Control and Epidemiology, Arlington, VA, USA; 4 Memorial Sloan Kettering Cancer Center, New York, NY, USA; 5 Ascension, Grosse Pointe Woods, MI, USA; 6 Cleveland Clinic Health System, Cleveland, OH, USA; 7 Duke University Medical Center, Durham, NC, USA; 8 Santa Clara Valley Healthcare, San Jose, CA, USA; 9 Atrium Health, Charlotte, NC, USA; 10 Children’s Hospital Colorado, Aurora, CO, USA; 11 LSU Health, Baton Rouge, LA, USA; 12 UT Health San Antonio, San Antonio, TX, USA; 13 University of Nebraska Medical Center, Omaha, NE, USA; 14 Beth Israel Lahey Health, Boston, MA, USA

## Abstract

The Society for Healthcare Epidemiology of America, the Association of Professionals in Infection Control and Epidemiology, the Infectious Diseases Society of America, and the Pediatric Infectious Diseases Society represent the core expertise regarding healthcare infection prevention and infectious diseases and have written multisociety statement for healthcare facility leaders, regulatory agencies, payors, and patients to strengthen requirements and expectations around facility infection prevention and control (IPC) programs. Based on a systematic literature search and formal consensus process, the authors advocate raising the expectations for facility IPC programs, moving to effective programs that are:

• Foundational and influential parts of the facility’s operational structure

• Resourced with the correct expertise and leadership

• Prioritized to address all potential infectious harms

This document discusses the IPC program’s leadership—a dyad model that includes both physician and infection preventionist leaders—its reporting structure, expertise, and competencies of its members, and the roles and accountability of partnering groups within the healthcare facility. The document outlines a process for identifying minimum IPC program medical director support. It applies to all types of healthcare settings except post-acute long-term care and focuses on resources for the IPC program. Long-term acute care hospital (LTACH) staffing and antimicrobial stewardship programs will be discussed in subsequent documents.

## Executive summary

Within all types of healthcare facilities, a wide array of preventable infectious risks that can lead to healthcare-associated infections (HAIs) exist. These can cause significant harm to patients, healthcare personnel, and visitors, and threaten the successful and safe operations of the healthcare facility. The facility’s Infection Prevention and Control (IPC) Program is essential to identify, mitigate, and prevent these infection-related harms, and over the past two decades, reductions in reported HAIs reflect an increased focus on HAI prevention and the requirement for facilities to systematically address these harms through their IPC programs. Despite these gains, IPC programs remain vulnerable and under-resourced, the composition of and resources committed to IPC programs vary widely, and the scope and intensity of IPC activities differ between facilities. Instead of being a foundational necessity that accommodates healthcare facilities of all types and sizes, an adequately resourced IPC program that addresses all infectious harms may be seen by some as optional and static. Given their impact on the safety, quality and operations of healthcare facilities, **the expectations for facility IPC programs must be raised**, moving to maximally effective programs that are foundational and influential parts of the facility’s operational structure, resourced with the correct expertise and leadership, and prioritized to address all potential infectious harms. This position paper from leading infection prevention and infectious disease societies outlines a call to action for healthcare facility leaders, IPC program members, and regulatory agencies. The role and impact of IPC programs, the components of IPC programs that are necessary to move from active to also being highly effective, and strategies to build towards a more effective IPC program are discussed.

## Introduction and call to action

***Healthcare-associated infections (HAIs) are common.*** In 2015, the U.S. Centers for Disease Control and Prevention (CDC) conducted their second national prevalence survey examining the burden of HAIs in U.S. acute care hospitals.^1^ Approximately 1 in 31 hospitalized patients developed a HAI after admission to the hospital. HAIs cause substantial morbidity and mortality and also have considerable impact on healthcare costs, length of stay, and the operational efficiency of a healthcare facility.^2–6^ Performance on select HAIs is now part of publicly reported quality measures with increasingly heightened financial penalties and inclusion in reputational consumer and publicly reported surveys,^7,8^ emphasizing the importance of preventing these harms as much as possible.

***Many other infectious risks exist in healthcare facilities***. While some HAIs are known to broad audiences due to publicly reported performance metrics (eg, bloodstream infections associated with central venous catheters used to deliver medication [known as central line-associated bloodstream infections, or CLABSIs]), other infectious risks exist in healthcare facilities that are absent from public HAI reporting. These include but are not limited to contagious disease exposures and outbreaks, breakdowns in device and instrument cleaning and sterilization/disinfection, lapses in environmental controls (eg, ventilation failures or water intrusions), and outbreaks related to contaminated medications, supplies, or medical products. These risks can lead to significant harm to patients, healthcare personnel (HCP), and visitors, and threaten the successful and safe operations of the healthcare facility.

***Reduction of these infectious harms is possible.*** Many of these infectious harms can be reduced through the application of recommended evidence-based practices (eg, hand hygiene, environmental cleaning and disinfection, standardized interventions around surgical and other procedures, safe medication handling, and interventions that mitigate the risk of patient- and HCP-related spread of contagious pathogens). Within healthcare facilities, it is essential that a rigorous program be in place to identify, mitigate, and prevent the wide variety of infection-related harms from affecting patients, HCPs, and visitors, as well as positively impacting the operations and the financial and reputational security of the facility. This program, typically known as the Infection Prevention and Control (IPC) program, is entrusted with having expertise around infection surveillance, control, and prevention; policy implementation; data analysis; epidemiology; regulatory requirements; and quality improvement to oversee and direct the efforts of the facility to prevent and control HAIs.

***IPC Programs are essential.*** The increased scrutiny and expectations by groups that assess the safety and quality of healthcare facilities have been important in raising the minimum standards of IPC programs to address harms caused by HAIs. IPC programs are required as part of the U.S. Centers for Medicare and Medicaid Services (CMS) conditions of participation (CoP). For acute care hospitals, CMS states:

*“The hospital must have active hospital-wide programs for the surveillance, prevention, and control of HAIs and other infectious diseases, and for the optimization of antibiotic use through stewardship. The programs must demonstrate adherence to nationally recognized infection prevention and control guidelines, as well as to best practices for improving antibiotic use where applicable, and for reducing the development and transmission of HAIs and antibiotic-resistant organisms. Infection prevention and control problems and antibiotic use issues identified in the programs must be addressed in collaboration with the hospital-wide quality assessment and performance improvement (QAPI) program.”* ^9^

Over the past two decades, reductions in reported HAIs reflect an increased focus on HAI prevention and the requirement for facilities to systematically address these harms through their IPC programs.^10^ Despite these gains, IPC programs remain vulnerable and under-resourced. This was illustrated during the COVID-19 pandemic when reported HAI rates increased significantly to levels not seen for several years as attention to core IPC practices eroded amid an overwhelmed healthcare system.^11,12^ The typical IPC program has the minimum resources to meet regulatory standards and reduce endemic infectious harms. IPC programs are often not resourced to mitigate and respond to unanticipated events, both facility-specific (eg, a unit outbreak of an infectious disease) and more regional or global (eg, a public health emergency such as the COVID-19 pandemic) while maintaining the hospital’s adherence to IPC practices for HAI prevention.

***“Active” does not necessarily mean fully effective***. Even in the absence of a global pandemic, the composition of and resources committed to facilities’ IPC programs vary widely, and the scope and intensity of IPC activities differ.^13^ While CMS requires an “active” IPC program, what constitutes active is not clearly defined. One facility’s IPC program that has an infection prevention committee with policy review, reports the required HAIs to CMS, and meets regulatory expectations might be deemed as “active.” However, compare that facility to the effectiveness of another, where in addition to the above, the IPC program partners with frontline staff in proactive practice assessments to reduce HAIs and track other infectious harms. Most importantly, the second facility conducts rigorous review of all HAIs for patterns of variability in expected practices, develops action plans for improvement, and sets and communicates institutional metrics and expectations for infection prevention for all its personnel. Comparatively speaking, the first facility may be “active” but not as “effective” as the second.

***IPC programs remain under-resourced and under-prioritized.*** The resources and support that facilities provide for their IPC programs are often extensively scrutinized and are constantly under threat.^13^ IPC programs are under-resourced and under-prioritized for a number of reasons, such as failure to add more resources to the IPC program in the setting of facility expansion (eg, through acquisition of ambulatory surgical or outpatient centers) and delegation of IPC leadership to individuals without the necessary competencies in infection prevention. These approaches reflect the perception that an adequately resourced IPC program that is able to address all infectious harms is optional and static, instead of being a foundational necessity that accommodates healthcare facilities of all types and sizes. Some facilities may only focus on publicly reported HAIs or IPC program functions that are expected by regulatory agencies instead of fully addressing the larger harms and activities that are not “required” but directly impact patient and facility outcomes. This under-resourced approach fails to recognize how the work of the IPC program is vital to the facility’s efficiency, effectiveness, and financial stability and neglects the larger burden of preventable harms. **It is time to raise the bar for IPC programs to ensure they are adequately and appropriately resourced, led, and supported.**

The Society for Healthcare Epidemiology of America (SHEA), the Association of Professionals in Infection Control and Epidemiology (APIC), the Infectious Diseases Society of America (IDSA), and the Pediatric Infectious Diseases Society (PIDS) represent the core expertise regarding healthcare infection prevention and infectious diseases (ID). Considering the subjectivity of the CMS requirement for an “active” IPC program and the threat of reduced resources and prioritization placed upon IPC programs, SHEA, APIC, IDSA, and PIDS have written this call to action for healthcare facility leaders, regulatory agencies, payors, and patients to strengthen requirements and expectations around facility IPC programs. **We advocate raising the expectations for facility IPC programs, moving to effective programs that are:**

- **Foundational and influential parts of the facility’s operational structure**
- **Resourced with the correct expertise and leadership**
- **Prioritized to address all potential infectious harms**

As noted by a survey of experts in the field, “[a]n effective [IPC] program is one that is “continuously improving the program, adopting new strategies to reduce the HAIs to the irreducible minimum’” and is focused on “proactive prevention and risk reduction.”^14^ This document discusses aspects of the IPC program, including the leadership and reporting structure, expertise and competencies of its members, and the roles and accountability of partnering groups within the facility. It applies to all types of healthcare settings, excluding post-acute long-term care, which will be addressed later in a partner document. This document’s recommendations for resources for the IPC program do not include recommendations for resources for a facility’s antimicrobial stewardship (AS) program. AS programs, which are important quality and safety programs that are often partnered with the IPC program, require their own resources and support to ensure effective impact. A forthcoming multisociety position paper will detail the recommendations on resource needs for AS programs.

## Intended use

This position paper followed the literature review process outlined in the “Handbook for SHEA-Sponsored Guidelines and Expert Guidance Documents.”^15^ No guideline, expert guidance, or position paper can anticipate all clinical situations, and this document is not meant to be a substitute for individual judgment by qualified professionals or to supersede more stringent state or regulatory requirements.

## Methods

This document follows the literature review and consensus process outlined in the “Handbook for SHEA-Sponsored Guidelines and Expert Guidance Documents.”^16^ The manuscript proposal was approved by the SHEA Publications Committee and the SHEA Board of Trustees.

### Literature review

The writing panel organized the document around several themes and within those developed PICO-style (population, intervention, control, and outcomes questions that were used in the development of search terms (medical subject heading (MeSH) and text word) by a professional medical librarian (see **Acknowledgements**). The librarian developed a search strategy for PubMed and Cochrane (January 2000 to April 2023), restricted to English language articles on human subjects. Panel members screened article abstracts. Using the abstract management software Covidence (Melbourne, Australia), the abstracts from the literature search yield were screened by two authors for inclusion or exclusion. Lead authors (TT and SW) resolved conflicts from the abstract screening, and author subgroups reviewed and extracted the remaining full-text articles for inclusion in the manuscript using a standardized form. See Supplementary Material, Appendix 1 for the literature review criteria, PICO questions, search strategies, and the Preferred Reporting Items for Systematic Reviews and Meta-Analyses.

### Consensus

This document was developed following SHEA’s process for reaching consensus,^15^ which includes anonymous comment and voting periods using an online voting form. For this document’s recommendations, full consensus was achieved.

### Authors

The authors include current and past members of the Society for Healthcare Epidemiology of America (SHEA). Rebecca Bartles, DrPH, MPH and Tania N. Bubb, PhD, RN served as authors and representative for APIC; Thomas G. Fraser, MD served as author and representative for IDSA; Vidya Mony, DO served as author and representative for PIDS. All authors and representatives served as volunteers.

### Review and endorsement

The document was reviewed and approved by the SHEA Publications Committee and endorsed by the SHEA Board of Trustees, APIC Board of Directors, PIDS Board of Directors, and IDSA Board of Directors.

## Section I: Infection prevention and control programs: essential role and multifaceted impact

*A core tenet of healthcare is to “do no harm,” but infectious risks to patients, HCP, and visitors are pervasive in all types of healthcare facilities. Much of this harm can be prevented through evidence-based infection prevention practices, such as hand hygiene, environmental cleaning, and protocolized handling of invasive devices. A core set of experts in these infection prevention practices who comprise the facility’s IPC program, can markedly reduce the risk and impact of infectious harms as well as the financial and reputational risk to the institution. Unfortunately, IPC programs at many healthcare facilities are under-resourced and may be seen as ancillary instead of foundational to successful facility operations. This section discusses the role of IPC programs and their impact on the operational success of all types of healthcare facilities.*

### Recommendation

*Healthcare facility leaders and regulatory partners should prioritize the expectation that facility IPC programs address all infectious risks and harms as a core requirement.*

An IPC program is a multidisciplinary and comprehensive prevention program that identifies, prevents and, if possible, eliminates the risk of acquisition and transmission of infectious pathogens and diseases—including HAIs—across the wide scope and complexity of the services provided within a healthcare facility. IPC programs address all facility types and sizes (including acute care, outpatient, ambulatory, and long-term care), patient populations, HCP, and facilities’ visitors. These programs aim to deliver cost-effective care to patients in a safe environment. Principal functions of the IPC program include (1) identification and surveillance of HAIs and other infectious harms, (2) setting policies and procedures that reduce the risk of these harms, (3) investigating and controlling infectious illness clusters and outbreaks, (4) utilizing microbial epidemiology and interventions to interrupt the transmission of infectious diseases, and (5) educating and training HCP regarding the principles and practices of IPC to support the development of a safe environment for all who enter the facility. Effective IPC programs use methods of surveillance, data analysis, and reporting based on accepted epidemiological principles, guidelines, and evidence-based research. They also use a collaborative, data-driven approach in developing and maintaining high-quality, coordinated programs of clinical patient care, education, investigation, and advocacy.

### IPC program regulatory requirements

Fifty years ago, IPC programs were underdeveloped additions to a healthcare facility’s operational structure. The value of such a program was unclear, and programs were noted as “cost centers,” given their lack of direct revenue generation. To address this concern, the landmark Study on the Efficacy of Nosocomial Infection Control (SENIC) in the 1970s first quantified the value of IPC programs, finding that facilities with robust IPC programs reduced HAIs by 32% (vs an 18% increase in hospitals without programs or with less robust programs).^17^ Post-SENIC, requirements for healthcare facility IPC programs increased.

The CMS CoP is a guiding regulatory requirement, utilized by accrediting bodies such as The Joint Commission, Det Norske Veritas, and others when assessing a facility’s level of safety and quality of care.^9^ Core components of the CoP include having qualified, trained, designated individuals serving in a leadership capacity; systems in place for HAI prevention, control, and monitoring; collaboration with quality assessment and performance improvement programs; and training systems in place for HCP. The healthcare facility’s governing body is responsible for the implementation, performance, and sustainability of the IPC program and provides resources to support and track the implementation, success and sustainability of the program’s activities.^18^ The subjective language in parts of the CoP (ie, requiring an “active” program) may lead to disparities in resources, prioritization, expectations, and activities of IPC programs across facilities.

### Impact of IPC programs

***The effective IPC Program:*** Patient harm events are often preventable and are the most common measure of success of an effective IPC program. Such programs monitor and report selected harms, like publicly reported HAIs, and actively reduce other infectious harm events. Effective IPC programs use tools such as the electronic health record, data analytics, proactive clinical practice assessments, and community infection incidence to continuously monitor the risk of infection and the occurrence of HAIs at the facility. These activities allow for timely dissemination of that information to facility leaders and frontline staff. Effective IPC programs have numerous positive effects on the healthcare facility including:

1. ***Reductions in healthcare-associated infections:*** IPC program work addressing HAIs in healthcare settings has led to important reductions in the core infectious harms (eg, reported HAIs). Implementation and adoption of evidence-based practices combined with systematic audit and feedback of performance has been shown to reduce key HAIs like CLABSIs and catheter-associated urinary tract infections (CAUTIs).^19,20^ Surgical site infections (SSIs) have been reduced through a bundled approach to care, including appropriate aseptic technique and preoperative antibiotic prophylaxis. Full implementation of these prevention interventions requires members of the IPC program to partner on educating clinical teams and auditing practices to address gaps in processes. The first decade of public reporting of selected HAIs saw marked reductions, including a nearly 50% decrease in CLABSIs, 35% decrease in CAUTIs, and significant reductions in other HAIs.^21^
2. ***Infectious risk assessment of operational system failures:*** While the IPC program’s work on reported HAIs may garner the most visible attention of healthcare facility leaders, an effective IPC program has a much larger impact on the facility through activities that assess and address the risk of other potential infectious harms. Activities such as instrument sterilization and device reprocessing audits, environmental cleaning compliance assessments, infection-related risk assessments of facility construction projects, facility water management program advisory, and hand hygiene performance tracking all identify gaps in care that could potentially lead to harm. Effective IPC programs also identify emerging risks shared via external advisories, regulatory alerts, and updated practice guidelines. While not captured as part of public HAI reporting, these activities reduce risks to patients, HCP, and visitors and help facilities avoid potential regulatory and financial penalties if such system failures lead to harm.
3. ***Reduction of waste/overuse:*** The diagnosis and treatment of infectious diseases can be fraught with inappropriate resource utilization. From the use of diagnostic testing in patients with a clinical picture that has a low likelihood for an infectious illness to specimen collection practices that lead to false positive results because of contamination, this variability can affect the quality and cost-effectiveness of care and also result in unnecessary and potentially harmful treatments. IPC programs have a leading role in diagnostic stewardship,^22^ improving appropriateness of test utilization, specimen collection, and interpretation while reducing waste and overuse.^23–26^
4. ***Protection of the HCP workforce:*** IPC programs play an integral role in protecting the facility’s workforce from infectious harms such as respiratory viruses and tuberculosis. Contagious pathogens often lead to workforce absenteeism and subsequent understaffing that may affect patient care. IPC programs monitor known and emerging infectious risks, perform surveillance for infections, and monitor compliance with isolation and other prevention practices in the facility, helping to swiftly identify and isolate patients with potential infections that may lead to additional cases of healthcare-associated illness. In concert with occupational health, monitoring of HCP absences and mitigation plans that include infection prevention measures (such as HCP vaccination) can keep HCP healthy and available to provide patient care through times of increased community circulation of infectious diseases.^27,28^ Conversely, infected HCP may work while ill (“presenteeism”), spreading disease while negatively impacting other measures of productivity and safety. IPC program members can champion efforts to reduce presenteeism and minimize this risk to others.^29–31^
5. ***Positive impact on core operational metrics:*** Effective IPC programs can help an organization achieve core operational goals. Organizations have measures of success used to hold their leadership team accountable for efficient and safe care. Many of these metrics are affected by patient harms, specifically HAIs, and can be positively impacted through the work of the IPC program. Examples of metrics enhanced by reducing infectious harms include length of stay, serious safety events, and readmissions.
6. ***Improvement of financial and reputational metrics:*** In addition to operational metrics, IPC programs can have significant positive financial and reputation-based impacts on the organization. HAI reductions can lead to reduced costs of care per patient discharge, and the savings from having an optimized IPC program targeting HAI prevention has been estimated at up to $13,000 per month for a hospitalized patient and up to $174,000 in critical care patients.^32,33^ In a study from one community hospital that implemented core infection prevention and safety practices, device utilization ratios, CAUTI, and CLABSI rates significantly declined resulting in an estimated cost savings of $688,050.^34^ Hospitals can also incur financial penalties through CMS’s value-based programs when HAI rates are higher than national goals. Eighty-three percent of the CMS’s Hospital-Acquired Conditions program penalty are specific to HAIs, including CLABSI, CAUTI, selected SSIs, and selected bacterial infections.^35^ In addition, high rates of infections may increase a facility’s risk of losing 2% of the withheld CMS payments under their value-based purchasing program.^36^ Other payors also include similar value-based programs where there is risk and reward for performance. These metrics have a component of mortality, harm, and readmissions which can all be linked to infectious harm events.
  Effective IPC programs decrease HAIs and improve patient-centered outcomes, impacts that will also be reflected as improvement in quality-based measures. Externally facing facility ratings for quality and safety, including employer-sponsored (eg, the Leapfrog Group) and consumer-focused (eg, *U.S. News and World Report*)^37^ programs, are also influenced by a facility’s performance on preventing select HAIs. While these surveys do not specifically include direct financial penalties for inadequate quality and safety performance, they do allow for incentives based on HAI rates in addition to potential impacts on a facility’s reputational standing.^38^
7. ***Impact and influence on the larger community:*** Healthcare facilities do not exist in isolation but as a part of their larger community. They often function as the epicenter of the community, offering supportive services like health fairs, health education, housing, transportation, and access to food.^39,40^ IPC programs within these organizations can impact the health status of the larger community through information and data sharing, public health partnerships, and community interventions and initiatives. IPC programs also have a primary role as a connector with local and state public health departments for early identification of infection outbreaks and support for outbreak control.

In summary, an effective IPC program that proactively addresses preventable infectious harms and risks can have marked positive impacts not only on the health and safety of patients, HCP, and visitors but also on the care financial, reputational, and efficiency outcomes essential to facility operations. IPC programs are critical to the facility infrastructure and must be considered as foundational and essential programs for the facility, regardless of size or populations served. Finally, the activities of the IPC program should be more than “active” and instead strive for maximum “effectiveness” to address and minimize all infectious harms.

## Section II: Necessary resources and components of an effective IPC program

*As IPC programs are necessary and essential parts of all types of healthcare facilities, the specific components of and resources provided to these programs are extremely important. Ensuring a leadership model that is most effective to advance the mission of the IPC program, a core set of trained personnel who can conduct these functions, and a clear and accountable reporting structure are vitally important to achieve an effective IPC program that reduces harm and improves the quality of care. This section outlines the components of IPC programs that are necessary to attain this goal and to move these programs from “active” to also being “effective.”*

### Core IPC program roles

Successful prevention of HAIs requires a multidisciplinary approach matched with the correct expertise to guide prevention efforts. There are two core roles within IPC programs that reflect this approach: infection preventionists (IPs) and IPC program physicians. IPs are specially trained professionals, leaders, educators, and collaborators who facilitate, lead, and promote efforts to reduce infections within healthcare.^41^ While historically, the majority of IPs came from a clinical nursing background, now these specialists have a diverse array of relevant career experiences, including quality improvement, public health, microbiology, and others.^14,42^ The diverse backgrounds of IPs benefit the expansive roles and duties of the profession due to its complexity and multifactorial components,^43^ incorporating all aspects of the healthcare arena – clinical, operational, regulatory, and administrative.

Some IPC programs have also included one or more physicians as part of their personnel. Highlighted as an essential part of an effective IPC program by the SENIC study,^17^ such physicians (also known in some facilities as “hospital epidemiologists”) are specialists who have added training in IPC. However, the term “hospital epidemiologist” can be confused with the role of epidemiologist in a broader, largely public health setting, in which individuals are often PhD or DrPH prepared versus physicians with medical and ID or other specialty training. Sounding more like an optional academic or research role, the hospital epidemiologist might be mistaken by facility operational leaders as solely an epidemiologic researcher rather than what is intended as a clinician with expertise in operational IPC. Such confusion may lead to insufficient resources and support for the role. Arguably, a term that better defines that individual’s expertise, competencies, and oversight using more operational terminology, such as the “Medical Director” for infection prevention, discussed in detail below, may more effectively communicate the essential role of these experts to facility leaders. Many IPC physicians have a specific background as ID physicians, which is well suited for the overarching goals of an effective IPC program.^44^ Experiences based on the practice of medicine combined with an understanding of clinical infectious diseases are valuable and may be difficult for those with other backgrounds to approximate, as most such training is primarily in the context of a formal ID fellowship. The training that ID physicians receive on the clinical presentation, management, and pathophysiology of infectious syndromes is an important competency that complements the skills and expertise of other members of the IPC team.^44^

Both of these core roles are fundamental to reaching the goal of effective IPC programs. While the degree of effort, dedicated time, and support necessary for each role may differ between various facility types, having each role as part of an IPC program should not be optional. For example, in smaller facilities, the physician role may only require a percentage of an individual’s effort and support to fully meet the expectations of a robust effective program. What is essential is that such roles are not only present but are held by competent IPC professionals and are supported appropriately for the volume and level of complexity of the healthcare facility they serve.

### IPC program leadership

#### Recommendations

1. *Healthcare facility administrators should support and resource a dyad leadership model for the facility’s IPC program that includes both physician and infection preventionist leaders.*
2. *IPC program leaders should have access to senior facility executive leaders that provide prompt support for the deliverables they champion.*
3. *Regulatory agencies and other evaluators of healthcare facility quality should examine IPC program leadership, including resource support, member competencies, and the program’s leadership model (including presence of both members of the leadership dyad), as part of their surveys of healthcare facility IPC programs.*

#### The dyad leadership model

Leadership and the integration of the IPC program throughout the healthcare facility have been cited as one of the most influential factors for an effective program in infection prevention.^14^ IPC program leaders must set and articulate goals and priorities, understand multiple, complex clinical and operational workflows, lead epidemiological investigations and analyses, and communicate and influence interested individuals and parties in multiple facilities to successfully implement processes to prevent HAIs and their associated harms.

Across U.S. healthcare facilities, leadership structures for IPC programs vary.^13,41^ CMS requires all hospitals to designate an “infection control officer,” and in many cases, this individual is also the infection prevention leader. Leaders of IPC programs have at some facilities included healthcare administrators, clinicians (both with and without a background in ID), and nursing leadership as well as individuals with a specialized focus and training in infection prevention (either a physician or an IP). Some have included a single individual charged with the oversight of the program, who also may be responsible for conducting the day-to-day activities of the program. While these leader phenotypes bring different insights to the role, to meet the raised bar of an “effective” IPC program, the program’s leadership should be filled by those with expertise and competencies in infection prevention who are allotted adequate time and resources towards IPC activities.

Given the increasing complexity of healthcare systems and the shift towards value-based care, implementation of a dyad leadership model has been utilized in healthcare facilities^45^ to improve communication, collaboration, and successful attainment of institutional goals.^46^ This model involves two individuals in different professions with shared responsibilities and aligned goals.^47^ The literature surrounding the dyad leadership model in healthcare and its impact most often involves unit-based clinician-nurse^48–51^ and clinician-administrator teams.^50,52^ Dyad leadership structures in healthcare have been associated with improvements in communication and collaboration, staff turnover, and patient satisfaction, standardization of care; successful policy implementation and culture change; and improved physician engagement and culture.^46^ Successful dyad characteristics include clearly defined roles and aligned goals within the dyad; a norm of interprofessional communication within the dyad; external communication of the purpose of the partnership to improve clinical care; training in leadership, conflict resolution, and quality improvement; and sufficient institutionally supported time and effort for both members of the dyad to perform their duties.^45,53^

While there are no studies of the dyad model of leadership within IPC programs, the model lends itself nicely to effective IPC programs at all types and sizes of facilities. Deemed the most successful model for IPC leadership among a qualitative survey of experts in infection prevention,^14^ the dyad leadership framework opens communication across physician-administration and physician-nursing structures.^52^ Characteristics cited as leading to successful leadership dyads in clinical settings (eg, sufficient financial and institutional leadership support, effective communication) align with characteristics of successful IPC program leadership.^54,55^

#### The medical director of IPC

In clinical settings, physician leadership of hospitals and healthcare systems has been linked with better quality scores and reputational rankings.^56^ Inclusion of a physician in the leadership of IPC programs is anticipated to lead to similar benefits.^14^ Such a role is noted here as the “Medical Director of IPC,” but it has also been referred to by other titles (Box). The intent with the terminology “Medical Director” is to utilize a role that is commonplace and familiar within many healthcare facility governance structures in order to better communicate the function of this leader. While the individual’s specific title may vary by facility, the intent is that this role **directs** and **leads** the IPC program alongside the Infection Preventionist Director. These individuals may be employed members of the hospital medical staff or credentialed physicians with an affiliated private practice.^57^ In some facilities, these individuals have simply chaired the formal IPC committee that periodically approves IPC policies, reviews HAI performance and notes other standing facility reports. They also may have limited to no training in infection prevention and the necessary competencies for individuals in this role (see Table 1).^58^ However, an IPC program physician leader with such limited and minimal expectations, dedicated support for, and involvement with the day-to-day activities of the program is **not acceptable** to consider such a program “effective.”^59^

**Table 1.** The Society for Healthcare Epidemiology of America core competencies for infection prevention and control physician personnel

Role	Competency Area
**Epidemiologist**	Surveillance (for HAIs and other harms); data management analysis and visualization
**Subject Matter Expert**	Clinical management and diagnosis of infectious diseases, pathogen transmission, infection prevention and control, outbreak investigation, microbiology and clinical diagnostics, special populations, and non-acute setting
**Quality & Performance Improvement Leader**	Quality improvement science
**Healthcare Administrator**	Leadership skills, program implementation, assessment, and advocacy
**Outcomes Assessment Evaluator**	Outcomes assessment and evaluation
**Regulatory and Public Health Liaison**	Public health, emergency preparedness
**Clinician Educator**	Effective training, teaching

Adapted from Kaye et al.^59^

The Medical Director is usually a practicing physician, which is an important attribute to bring to the role, as a clinician’s insights into the practice of medicine and the nuances of patient care are critical to the development and implementation of IPC processes in the clinical healthcare setting. This perspective gives the Medical Director and the IPC program additional credibility with key influential frontline and administrative personnel, allows for this role to serve as a liaison with the medical staff, and facilitates implementation of changes in HCP processes and behaviors. As noted earlier, many IPC Medical Directors are ID physicians, who are well suited for this role. The physician component of the IPC program leadership is often forgotten in guidelines on IPC program structure. Notably, the CMS CoP, while requiring a “qualified, trained” leader of the IPC program, states this leader may be an IP or other “infection control professional” but does not specifically mention a physician leader.^9^ This gap also is present in requirements from agencies that survey and accredit healthcare facilities. The Joint Commission recently revised requirements for IPC programs among acute care facilities, but these also fail to specifically mention physician leadership as an essential part of the IPC program.^60^

#### The infection preventionist director

The other essential member of the IPC Program dyad leadership is the IP leader.^41^ While the role of infection preventionist is inherently a leadership role, it is critical to program effectiveness that a program have a designated IP leader with appropriate support and authority to ensure program activities occur as intended. Like the discussion above around the term “Medical Director,” the IP leader role here is termed the “Infection Preventionist Director” to note that this individual **directs** and **leads** the IPC program alongside the Medical Director, but they may be referred to by other titles (Box). Even small facilities that employ only a single IP require an IP leader to maintain responsibility over the day-to-day operations and strategic planning of the IPC program. While one individual may serve in both roles in such facilities, it is essential that that individual is empowered and has dedicated time and support to serve as such. The Infection Preventionist Director is responsible for the creation and execution of the facility’s IPC program evaluation, risk assessment, and plan, as well as all infection prevention-related policies and procedures. This individual is also responsible for ensuring all IPC program activities are conducted (eg, surveillance, rounding, practice gap identification, performance improvement interventions) and effectively communicating status and progress of program efforts with other facility leaders.

Box: IPC Program Leader Titles

**Medical Director of IPC:**

- Hospital Epidemiologist
- Healthcare Epidemiologist
- Physician Leader of IPC
- Clinical Leader of IPC

**Infection Preventionist Director of IPC:**

- Operational Leader of IPC
- Administrative Leader of IPC
- Manager of IPC

#### Supports and risks to successful dyad leadership

To attain an effective IPC program, the Infection Preventionist Director and Medical Director must work in partnership, providing complimentary and synergistic skills and competencies. Both the Infection Preventionist Director and the Medical Director should be considered members of the facility leadership team and should be included in multidisciplinary discussions and decisions in which infection prevention may be a factor. Together, these leaders set the strategic priorities for the IPC program and ensure alignment with facility goals, share critical information on IPC issues with senior and department leaders, and provide support for other IPC team members. The success of this dyad depends on many factors, including the organizational culture, interpersonal and personal attributes, and leadership abilities and expertise of the respective dyad leaders.^46^ Each person in the IPC leadership dyad must equally be supported by the organization and be supportive of the other leader’s expertise and contributions.

Co-leading a program can have its challenges, particularly if the goals of the two dyad leaders and the senior leaders to whom they report are not aligned.^14^ Additional challenges include preference of senior leaders or frontline HCP for one dyad leader over the other. This may be due to leadership style, experience, perceived knowledge, or professional background (eg, physician versus nursing). Due to implicit, explicit, and imbalanced power distributions between physicians and other non-medical professionals,^61^ however, the dyad leader relationship must be intentionally developed, with particular attention to avoiding diminishing either member of the dyad. Successful leadership teams will support each other publicly and clearly communicate shared and separate areas of expertise. Having different reporting lines for the leaders of the IPC program (eg, the Medical Director reports to the Chief Medical Officer while the Infection Preventionist Director reports through the quality program or nursing leadership) may bring ambiguity to decision-making, goal planning, and implementation strategies and can lead to confusion within the program.

Differences in protected time for their roles may also jeopardize the success of the dyad leaders. For example, the Medical Director may have significant clinical responsibilities that restrict the amount of time that is dedicated to IPC program work. This is especially a risk where the Medical Director is provided a limited amount of focused effort to support this role (eg, only support for a small percentage of salary or on a limited hourly basis that does not allow full involvement in the expected spectrum of activities of an effective IPC program). These physicians often appear as consultants rather than partners to the Infection Preventionist Director. Other threats to the dyad are related to Infection Preventionist Directors with additional responsibilities beyond strategic planning and managing the full IPC team, including acting in the roles of the IP team members covering clinical units and performing surveillance for HAIs. If the Infection Preventionist Director “counts” as a regular team member in the budget, they are often unable to hire additional staff to backfill their day-to-day role and responsibilities. These additional roles may cause the Medical Director to appear more available and “in charge,” having more dedicated time for IPC program oversight and work. Infection Preventionist Directors who are the only IP members of IPC team will manage all facets of the IPC program and may not have enough protected time to interact with facility leadership or strategically plan for the IPC program. These variations in team structure, leadership support, and role expectations affect how the dyad is viewed by leadership and colleagues and can impact the success of the program.

### IPC personnel competencies

#### Recommendation

*Healthcare facility leaders and regulatory partners should ensure that IPC program personnel are trained and have the core competencies outlined for these specific roles, including providing expectations and support for training when needed.*

Essential to the structure and effectiveness of the IPC program is ensuring that the members of the IPC program demonstrate the core competencies and skills necessary for their roles. For the two main groups of IPC personnel, physician and IP, there are previously outlined competencies (see Tables 1 and 2) that should be expected for individuals serving in these roles. In addition, such training should be ongoing throughout the individual’s career to incorporate the emerging knowledge and science around infection prevention. Importantly, some individuals hired into the IPC program may have a background or understanding around some of these competencies but lack a complete understanding and training in all the essential skills and competencies. For example, an ID physician may understand the clinical management of infectious diseases yet still needs further training in epidemiology and infection prevention practices to effectively function in an IPC role. Likewise, an IP with a nursing background will need training in other aspects of the IP role such as surveillance methodology, microbiology, and epidemiology. As such, facility leaders should set expectations for and support training for such individuals to attain the full range of these skills and competencies.

**Table 2.** The Association of Professionals in Infection Control and Epidemiology infection preventionist (IP) competency domains^63–66^

IP Competency Domain	Description
**Leadership**	Communication, critical thinking, collaboration, program management, mentorship
**Professional Stewardship**	Accountability, ethics, financial acumen, understanding of population health and continuums of care, advocacy
**Quality Improvement**	Subject matter expertise in infection prevention, performance improvement skills, data interpretation and analysis, facilitation of improvement programs, risk assessment
**Infection Prevention and Control Operations**	Knowledge of epidemiology and surveillance methods, IP rounding, education, cleaning/disinfection/sterilization, outbreak detection and management, emerging technologies, antimicrobial stewardship, and diagnostic stewardship
**Infection Prevention and Control Informatics**	Data management, analysis, and presentation

For physician IPC personnel, SHEA has outlined roles and associated competencies, knowledge expectations, and skills. These are briefly summarized in Table 1 but are outlined in detail in the position paper by Kaye et al.^59^ A certificate in healthcare epidemiology and IPC is also available through a SHEA/CDC training course. This course, or its equivalent, is taken by many IPC program physicians, and some facilities and states have required such training for their physician IPC personnel as a measure of IPC competency.

Likewise, APIC has developed a set of competencies outlining the essential knowledge and skills for an IP. This competency model was first described in 2012^62^ and has been updated (most recently in 2019) as the field has evolved.^63–66^ The model includes six domains meant to guide IPs in their career and leadership development (see Table 2). Subdomains are included in each domain which expand the specific skills, knowledge, and training required.^41^ IP specialty training typically occurs on the job; however, there are some academic programs that offer IPC degrees, diplomas, and certificates. Future academic pathways to the IPC profession are currently in development by APIC.^67^

IPs should obtain certification, through the Certification Board of Infection Control and Epidemiology (CBIC).^68^ Certification in Infection Control through CBIC “supports higher salary compensation, increases job satisfaction through a structured career development framework, improves patient outcomes, advances evidence-based infection prevention practices, and is valued by the public and within the health care industry.”^69^ Additionally, certified IPs may have a stronger understanding than other practitioners of the evidence for certain IPC practices and are more likely to recommend implementing such practices in their own facilities.^70^ Facilities should demonstrate that attainment of certification is a priority by dedicating appropriate time, resources, and support to IP staff to attain this level of expertise.

### Additional core IPC program team members

Other core personnel are also important to the goal of achieving an effective IPC program.^14^ These individuals may include persons who specialize in surveillance, data analytics/informatics, or process engineering. Facility leadership should ensure adequate resources are provided that include funding for these critical core members to achieve the goals of an effective IPC program.

#### Infection preventionists team members

In any IPC program, there is a need to have core members to conduct the key activities of the IPC program. Reporting to the Infection Preventionist Director, these individuals have similar core competencies and are the primary team members responsible for conducting HAI surveillance, partnering with frontline personnel to audit and train on expected IPC practices, facilitating IPC improvement programs, and conducting IPC risk assessments across many aspects of the facility. IP core team members may have a concentrated focus in specific populations (eg, assigned to areas and activities in a specific clinical area) or on specific IPC activities (eg, in a centralized HAI surveillance role).

#### Associate physician experts/epidemiologists

In addition to the Medical Director role, there is often a need, particularly for larger and more complex facilities, to include and provide support for additional physicians with IPC expertise on the team. This allows for concentrated focus in core areas (eg, a pediatric ID specialist to oversee the IPC program activities in pediatric units) as well as efficient and nimble physician guidance during times of unpredictable surges in IPC events or issues. As with the Medical Director, these individuals should have training in the core IPC competencies and be provided with adequate support and protected time to allow for a full focus on the IPC program activities and goals. The Medical Director should provide direct supervision, guidance for day-to-day activities, and mentorship to these colleagues. In addition to the physician and IP personnel, the IPC team, particularly in larger facilities, may also include individuals such as non-physician epidemiologists, who work with the IPC team, providing advanced expertise around key epidemiologic issues, assessments, and projects related to the program.

#### Data scientists

Data are essential in IPC program efforts, as they provide insights into program effectiveness and impact, identify new or under-controlled harms, and drive improvement. Data scientists serve to compile, analyze, and display data internally (eg, such as with efforts to display infection trends to frontline staff, institutional oversight committees, and leadership) and externally (eg, as is required by CMS, state and local health departments, and assessors of institutional safety and quality). These specialists can assist with large-scale data mining from available institutional sources like the electronic health record to aid with process improvement projects and epidemiologic investigations (eg, disease outbreak investigations).^71^ With the advent of artificial intelligence (AI) and machine learning and their uses by IPC programs, these specialists will be increasingly important for the IPC program mission.

#### IPC practice auditors

One key distinction of an effective IPC program is the proactive and comprehensive assessment of IPC practices throughout the facility, often completed through direct audits and observation of practice (eg, hand hygiene observations and environmental cleaning audits). To fully perform this function, some IPC programs, particularly those at larger facilities with a more complex range of patient populations and services provided, will need personnel dedicated to auditing frontline practices. They can also play a key role in real-time feedback and education to frontline personnel on core IPC expectations.

#### Administrative support

As with any other foundational facility program, the IPC program must be provided with administrative support to manage the important logistics of the IPC team. These include but are not limited to scheduling, office maintenance, and documentation of meeting activities. These core personnel will allow the other IPC team members to operate at the top of their expertise to lead and run an effective IPC program. In some instances, this role may need to be expanded into the role of a program manager who can coordinate the activities of a core component of the IPC program (eg, an individual who coordinates the institutional hand hygiene program).

### IPC program components: staffing to attain an effective program

#### Recommendation

*The IPC program, including its leaders, should be staffed and supported to allow ample time for completion of all IPC responsibilities.*

The SENIC study noted the impact of a better-resourced IPC program on the control of HAIs.^17^ That study demonstrated that the presence of one “infection control nurse” (ie, an IP) per 250 beds, a physician dedicated to the IPC program, and a system of reporting infection rates to surgeons was associated with a 32% decreased infection rate. The size and complexity of hospitals and healthcare systems have increased substantially since this seminal study. A later analysis from a large teaching hospital observed 1.6 million patient days of HAI surveillance and determined that increased IP staffing and the presence of an IPC program physician were associated with lower rates of HAI outcomes.^72^

Assessments of and recommendations for staffing of an IPC program are limited and often are focused on IPs. In 2018, the APIC MegaSurvey noted a median IP staffing rate of 1.25 IPs per 100 inpatient census beds.^73^ There were significant differences in IP staffing, duties, and support between smaller and larger facilities. With the advanced complexity of patient populations and healthcare procedures, including an expanding ambulatory scope of healthcare, ***use of occupied or licensed bed size as the sole metric to assess staffing is no longer useful and underrepresents the resource need for an effective IPC program***. A report from 2002 recommended that, in addition to facility bed size, the scope of the IPC program, complexity of the health care system, characteristics of the patient population, and the unique needs of the facility and the community must also be considered.^74,75^ A large nonprofit health system systematically quantified IP staffing needs, including in acute care settings and continuum of care sites such as ambulatory, long-term care, and home health. They determined a benchmark of 1 IP full-time equivalents (FTE) per sixty-nine beds using this approach. The authors emphasized that the scope of services, population, as well as size of the facility must be considered in determining IP staffing needs.

While formal recommendations about Medical Director and physician staffing remain rare, the SENIC study noted the presence of a trained physician as part of an effective IP program. An infrastructure report from members of SHEA in 2016 included recommendations for physician support that included considerations of the complexity of services provided and facility size.^76^ For academic-based institutions, hospitals with ≥300 beds and/or ≥50 intensive care unit beds, ≥ 1.5 FTE of a physician full professor salary was recommended, and for hospitals with < 300 beds and/or < 50 intensive care unit beds, ≥1.0 FTE of physician salary was recommended. For community-based hospitals with ≥300 beds and/or ≥50 intensive care unit beds, >1.0 FTE of physician salary was recommended, and for hospitals with <300 beds and/or <50 beds, ≥0.5 FTE of physician salary was recommended. As with the earlier recommendations for IP staffing, these guidelines did not fully account for the complexity of services provided, patient population, or larger continuum of care.

The Department of Veterans Affairs released a directive in 2017 (amended in 2021) that outlined the minimum staffing requirements for their facility’s IPC programs, including both the Infection Preventionist Director and Medical Director roles. They note the requirement for both an “Infection Prevention and Control Professional” (ie, IP) and a “Hospital Epidemiologist” (ie, physician that is “preferable to be an infectious diseases trained physician”) as part of the program. Importantly, they specifically note that “[t]he responsibilities for hospital epidemiolog[ist] are independent of clinical workload,” highlighting importance of providing enough dedicated time towards the IPC program to conduct the program work. Based on the facility complexity, there are levels of support for core IPC personnel as well as other requirements for the IPC program, including space and information technology and data analytic access.

In 2016, the World Health Organization (WHO) released expected core components of facility IPC programs with updated assessment tools released in 2023.^77,78^ These include expectations on IPC team members, objectives, and functions; IPC education of HCP; HAI surveillance; HAI improvement interventions; audit and feedback of IP practices; environmental controls; and facility staffing and bed occupancy. Notably, while the WHO recommends having a “dedicated, trained” team in place as part of the IPC program, specific expertise and leadership of the program is not noted nor is there an explicit mention of the need for physician leadership over the program. The WHO report recommended a minimum ratio of one full-time IP for every 250 beds but also noted that a higher ratio (eg, 1:100) should be considered. The 2023 assessment tools are stratified by facility type (primary, secondary, and tertiary) and note the same expectation for trained IPs (with ratio of one full-time IP:250 beds again noted for secondary and tertiary facilities), yet IPC leadership and reporting structures are not addressed.

Given the importance and impact of IPC programs, it is surprising that there are limited guidelines or requirements around IPC program composition, leadership, and support. With many studies focused only on inpatient bed size as the measure of the population at risk for infectious threats at a healthcare facility, resource statements on IPC staffing underestimate the true need, particularly with the raised expectations for a fully effective program. To guide facilities on an assessment of the resource needs to support an effective IPC program, APIC has created a staffing calculator that accounts for the healthcare facility’s size, practice type, patient population complexity (eg, pediatric, immunocompromised, and trauma patients) and services provided (eg, transplantation, congregate living).^79^ This tool provides a recommended range of IP staffing support.

We have also crafted a framework tool with recommendations around levels of expected support for the IPC Medical Director and other physician personnel (as applicable). This framework accounts for differences in facility volumes, demand, and complexity that would warrant a greater level of physician support to fully direct and address infection prevention issues in increasingly complex and busy centers (Table 3). Future versions of the APIC calculator will also expand to include recommendations on physician (using this framework in part) and other core member personnel support to guide facilities and other partners on expected staffing for IPC programs.

**Table 3.**
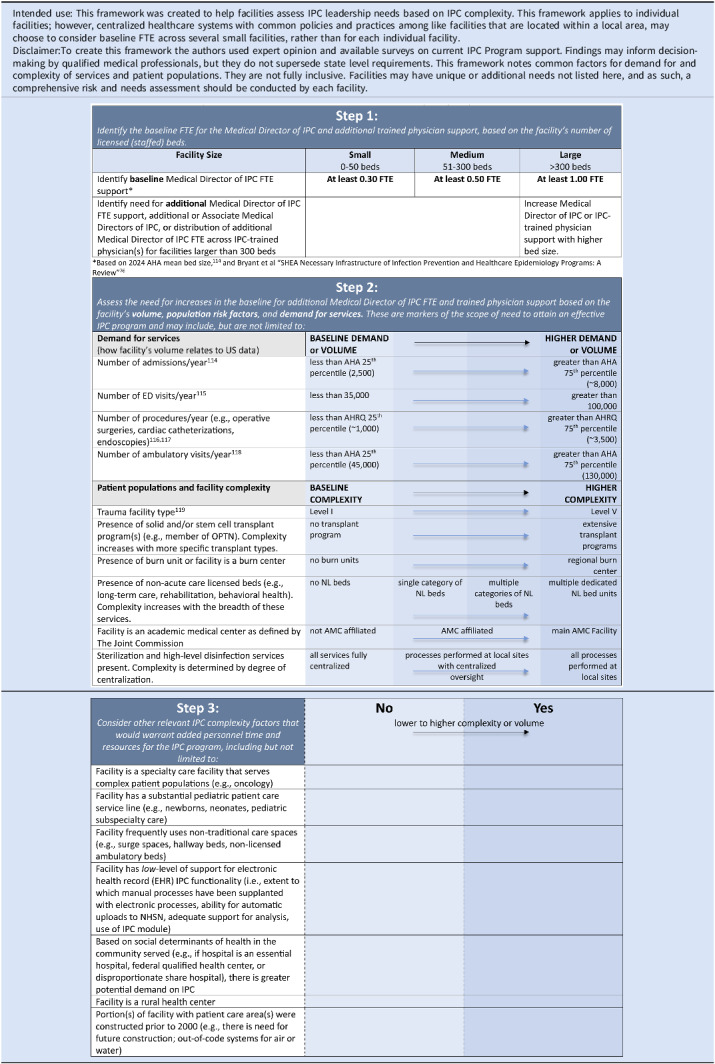
Medical director of infection prevention and control (IPC) and IPC physician support recommendations

FTE = full time equivalent; AHA = American Hospital Association; AHRQ = Agency for Healthcare Research and Quality; OPTN = Organ Procurement and Transplant Network

Example application of this framework: A 250-bed facility examines IPC physician support by starting with the recommended baseline support in Step 1 (0.50 FTE or equivalent). Based on the facility’s volume (i.e., if it is lower or higher than national 25th/75th percentiles respectively), complexity of the population served, or other factors noted in Step 3, the baseline support may need to be increased (e.g., through increased funding of the single Medical Director or addition of more IPC physician personnel) to fully meet the expectation of an active and maximally effective IPC program.

Multiple factors must be considered in determining baseline levels of support for IPC program Medical Director and other physician roles. As highlighted above, literature on IPC program staffing traditionally has been solely focused on the IP role, so any current recommendation for physician support is going to be largely based on expert opinion, as was used to set the recommended baseline support ranges in the framework table (see Supplementary Material, Appendix 2). In addition, many facilities may not currently support their IPC physicians with the minimally recommended levels noted in the framework. This is not unexpected, as the driving goal of this paper is to advocate for IPC programs to maximize their effectiveness and address all infectious harms. Facilities with under-supported IPC physicians may not have set expectations for these program members that fully align with a maximally effective IPC program, and they will need time to provide increased support and expectations around their IPC program personnel. It is also imperative that IPC physicians have dedicated time to commit to the breadth of expectations of their role and to avoid creating conflicting demands between physician clinical work and effort devoted to the IPC program. Such support provides the consistent presence and perspective of the IPC physicians that may be lost with more sporadic support for IPC program participation.

### IPC program: key partnerships and collaborators within the healthcare facility

#### Recommendation

*Facility personnel not directly supported under the IPC program but whose roles and activities impact and affect the mission and goals of the IPC program should have alignment of their performance goals that include partnership with and focus on IPC activities.*

Creating a fully effective IPC program involves partnerships with personnel from other key facility departments and entities. These personnel have expertise and roles that can directly impact the effectiveness of the IPC program, both positively, if their work aligns with IPC goals, and negatively, if the goals and priorities of their program run counter to those of the IPC program. For example, a facility’s environmental services (EVS) program that places priority solely on rapid and efficient room turnover could directly reduce the effectiveness of the IPC program if such rapid turnover does not allow for adequate time to clean the patient rooms thoroughly and reduce the risk of transmission of contagious pathogens. The support and resources for these partner personnel are budgeted separately from the formal IPC program. As such, to have as effective an IPC program as possible, healthcare facility leaders should set expectations for each of these partners that includes alignment with IPC goals and appropriate resources to meet those goals. Several key partners and their specific influence upon IPC goals are noted below:

#### EVS personnel

Pathogens can survive in the environment for prolonged periods of time, and EVS personnel play a key role in preventing infectious pathogen transmission in the facility. Their work in ensuring regular cleaning and disinfection of surfaces, equipment, and the facility environment reduces the risk of transmission of infectious pathogens throughout the facility.

#### Occupational health (OH) personnel

HCPs are subject to a myriad of infectious disease risks in the day-to-day care of patients. A facility’s OH program and personnel are responsible for ensuring a safe workplace, and partnership with the IPC program can effectively monitor and address potential infectious disease harms for HCP. Specific examples include evaluation and audit of personal protective equipment use when caring for patients in isolation precautions, adherence to Occupational Health and Safety Administration requirements to reduce bloodborne pathogen exposures, implementation of interventions to reduce the presence of infectious persons in the facility (whether visitors or other HCP), and oversight of the facility immunization programs for both HCP and patients.

#### Microbiology and laboratory personnel

Many HAIs are identified by testing and processes centered in the microbiology laboratory. Laboratory personnel impact IPC programs through their work towards the prompt detection of infectious organisms, often using newer molecular methods for diagnosis and surveillance, which allows for early identification and isolation of colonized or infected patients. Microbiology colleagues also play a crucial role in outbreak management through rapid identification of cases, molecular sequencing to determine relatedness of possible clinical cases, and partnering with state and referral labs if needed for additional testing or environmental cultures to identify a common source for the outbreak. IPC program members also partner with laboratory leaders on diagnostic stewardship programs to optimize the assessment of patients with suspected or confirmed infectious diseases while minimizing false positive results and associated waste and overuse (eg, unnecessary testing and treatments).

#### Industrial hygienists

These specialists play important roles to ensure a safe and healthy environment for patients and HCP through assessments of the environment and implementation of measures such as engineering controls (eg, improve ventilation, install physical barriers) to help mitigate spread of infectious pathogens. They work closely with the IPC program to stay up to date on industry-specific standards, assess risk and assist with outbreak response, if needed.

#### Informatics/Information technology specialists

Much of the work of the IPC program involves rapid detection of HAI activity increases and assessments of provider compliance to core infection prevention practices. Facility informatics specialists leverage the electronic health record and other data systems to aid IPC activities, such as through the development of tools for automated surveillance for HAIs, decision support interventions that decrease unnecessary laboratory testing, communications for contagious disease notification, and rule-based alerting and automatic ordering of key IPC actions (eg, automated placement of patient isolation orders when a resistant organism is detected).^80,81^

#### Emergency management personnel

The importance of these experts has been highlighted by experiences with recent outbreaks and pandemics such as those related to Ebola Virus Disease and COVID-19. Their expertise synergizes with that of the IPC program personnel, with an aligned goal to reduce the spread of novel infectious pathogens in the facility. They work to assess risks, establish communication protocols, facilitate coordination with various key parties (eg, community organizations, public health agencies), streamline and address supply chain vulnerabilities and shortages (eg, for medications, protective equipment), and direct resources to areas of most need. With the recently revised requirements from The Joint Commission that include two new elements of performance to enhance facility preparedness for high-consequence infectious diseases or special pathogens, partnerships between IPC programs and emergency management personnel will become even more important.^60^

#### Supply chain personnel

Individuals who manage the facility’s inventory of supplies are key partners with the IPC mission. Working with the IPC program to identify products that reduce infectious risks (eg, safety sharp devices) as well as whether products espoused to lead to HAI reductions (eg, antimicrobial coated devices) are necessary for the facility to meet IPC goals. Other examples of the supply chain IPC partnership include responding to product recalls due to pathogen contamination and standardizing facility disinfectants and hand hygiene products.

#### Sterile processing and device reprocessing personnel

The risk of pathogen transmission from inadequately sterilized or reprocessed devices is a growing concern that can lead to direct patient harm and substantial financial and reputational harm to the facility.^82–85^ Issues related to improper instrument sterilization and device reprocessing were a leading source of facility citations by The Joint Commission in 2023, including immediate threats to patient safety that can result in substantial facility penalties.^86^ Linkage of the goals of the IPC program with those of the sterilization and high-level disinfection programs is key to reduce infectious risks to patients, ensure standardized performance of sterilization and reprocessing practices, and minimize institutional risk.

#### Implementation experts

These specialists often have a background in quality improvement with specific training in a variety of improvement strategies (eg, LEAN, Six Sigma), and work to ensure that institutional policies, including those specific to the IPC program, are implemented effectively.^76,80,81,87–89^

### IPC program reporting structure

#### Recommendation

*The IPC program and its dyad leaders should have an organizational structure that allows for alignment of duties and responsibilities.*

The effectiveness of an IPC program is influenced by its reporting structure, affecting communication, collaboration, and the implementation of preventative measures. Of critical importance for the IPC program is ensuring direct reporting or regular access to an executive who will provide prompt support for IPC projects and policies.^48,52,64,71,90–92^ There is no national standard for IPC program structure, however, with some programs directly reporting into the healthcare quality department, while others through nursing or physician leadership. The dyad leadership relationship needs to support IPC-related clinical, quality and safety initiatives, reduce barriers with IPC practice and policy implementation, and achieve core outcomes in quality and safety.^48^ Such work will be more successful in the presence of organizational alignment. Importantly, irrespective of the IPC program reporting structure adopted at the facility level, it is important to have alignment in reporting for both dyad leaders.

With the multidisciplinary nature of engagement of the IPC program, a common reporting structure is through the quality department. Reporting to the institutional quality leader, such as the Chief Quality Officer (CQO), offers several advantages. The CQO is responsible for data-driven decision-making and performance measures across the health care organization.^71^ By reporting to the CQO, as long as that individual is part of the facility’s core leadership with the ability and expectation to direct and influence institutional practices and policies, IPC efforts may be better aligned with the organization’s strategic goals and have appropriately allocated resources. The IPC program will have easier access to relevant data and analytics resources, facilitating evidence-based decision-making. This support is essential for tracking infection rates, identifying trends, and implementing targeted interventions. Certain root causes of higher HAI rates, such as the frontline staff’s ability to perform expected practices consistently and reliably, can also lead to other safety harms which would be under the CQO’s purview. Furthermore, as IPC teams are closely tied to regulatory requirements and accreditation standards, the CQO typically oversees compliance efforts. With a unified reporting structure through quality, the approach will be more coordinated to meet regulatory standards and reduce the risk of noncompliance and potential penalties. Due to the natural synergy between the IPC and quality programs, it seems reasonable to have an IPC team report into the quality structure to contribute best to a culture of continuous process improvement.^48,52,64,71,90–92^

### Expectations for facility administrators

A 2008 collaborative report from the Betsy Lehman Center for Patient Safety and Medical Error Reduction, JSI Research and Training Institute, Inc., and the Massachusetts Department of Public Health noted that “[w]hile the [IPC] program must guide the effort, reducing the risk of HAIs is a hospital-wide responsibility, requiring teamwork and a multidisciplinary approach. Preventing transmission of infectious agents must be a hospital priority and part of institutional objectives.”^93^ For a facility IPC program to be effective in addressing and minimizing all infectious harms, the facility’s operational leaders also have a core responsibility and role. Leaders of the facility must set active expectations that IPC practices and policies are followed, set accountability of all personnel for IPC practices, and direct adequate resources to support the IPC program activities and mission. Similarly, an effective IPC program provides facility leadership actionable data with recommendations to prevent healthcare-associated infectious morbidity. At a facility with higher than desired HAI rates, outbreaks of infectious pathogens, or failures of core safety or infection prevention, all leaders, providers, and support teams including the IPC program share the responsibility for improvement.

## Section III: Applying recommendations to attain an effective program and strategies to optimize available IPC program resources

*The recommendations in Section II are intended to raise the bar and move all healthcare facilities to the goal of active **and** effective programs. For some facilities, applying these recommendations may initially be challenging. In addition, many facilities may not currently meet the criteria outlined above for the structure, personnel, and resources necessary for an effective IPC program but should work towards achieving this goal. As work commences to strengthen the IPC program, facility and IPC leaders need guidance on ways to move towards a more effective IPC program. This section will discuss in more detail how to apply the recommendations to a variety of facilities as well as strategies to build towards a more effective IPC program.*

Currently, facilities may not meet some of the recommendations that raise the expectations for IPC programs to be maximally effective. Facilities with a non-dyad leadership structure, with no partner physician personnel, with IPC personnel that have not been fully trained in the IPC competencies for their roles, and with limited access to specialized expertise may initially struggle with how to apply these recommendations to their facility. The intent of this document is to push the multitude of core groups who influence the prioritization, resources, and expectations around IPC programs (eg, facility leaders, regulatory agencies) to raise the bar for these programs while realistically recognizing that attaining this goal will take time, resources, and culture change. Implementation of these recommendations, however, is intended to be adaptable to address the specific attributes and risks of a given facility. For example, while the dyad leadership model is an essential expectation for all facility IPC programs, this does not mean that every facility must support the full salary for both individuals. Smaller facilities may be able to fully perform the spectrum of activities for an effective program with these roles receiving partial support. What is essential is that the support allows for full attention to and completion of these activities with dedicated time and effort.

To guide IPC program and facility leaders, we have provided guidance specific to aspects of IPC programs and facilities that may not, at first review, meet the expectations outlined for an effective IPC program (Table 4). Outlined are example strategies to ensure that members of the team provide the full scope of expected competencies for the IP and physician roles, which allows for synergy between leaders in other facility roles, and that sets the expectation that ample dedicated time is allowed for IPC team members to perform their roles fully and effectively.

**Table 4.** Application of the infection prevention and control (IPC) program dyad leadership model in different types of healthcare facilities and IPC program scenarios

Facility/IPC Program Example		Examples of How the Effective IPC Program Recommendations Can be Applied
***Aspects of IPC Program Leadership***	**Program has a dyad non-IP Medical Director who is not a physician**	Such leaders (e.g., with an epidemiology background) bring several key and important skills and competencies to the role but may be lacking a background in clinical medicine. Such leaders can be partnered with a physician with such insights (e.g., an infectious diseases [ID] physician who can serve as an associate leader) to ensure the full set of competencies for the physician leader role are met. Facilities should support and resource such partnerships so that the dyad leadership can have the full scope of skills and competencies necessary to maximize program effectiveness.
	**Program has a dyad Infection Preventionist Director without a nursing background**	Similarly, an Infection Preventionist Director without a clinical background (e.g., those coming from a public health or laboratory background) also bring some core skills to the role but may need resources or added personnel to help ensure that all core competencies are present in the IPC program. This could include set expectations for clinical rounding and shadowing and/or added IP personnel with such backgrounds.
	**Program has a physician member of IPC program who is not trained in IPC competencies**	IPC programs with non-IPC trained physician involvement (e.g., a clinician who chairs the IPC Committee) can take several steps to move their program to the expectations for an effective program. Such physicians should have expectations and resources to undergo training in IPC. In addition, they should have expectations and supported dedicated time to have a more active role in the program, beyond serving as a committee chair but instead as an active and engaged member of the team.
***Aspects of the Healthcare Facility***	**Small (<50 bed) community acute care hospital**	At a smaller facility, the resources and staffing necessary to attain an effective program may still be small (e.g., one IP). In this instance, several steps can align such a program with the expectations noted in this paper. The IP should have enough time to serve as the strategic dyad Infection Preventionist Director of the program as well as conduct the day-to-day IP activities. In addition, a physician partner for the program should be identified, such as the Chief Medical Officer or Chief of Staff. In this role, these individuals should have expectations for added training in IPC, such as through the SHEA/CDC Training Course, and also need to have dedicated time and expectations devoted to IPC program in an active and engaged manner.
	**Large acute care hospital**	Larger facilities may also have a higher complexity of services. As such, it is essential that the IPC program not only have dyad leaders that are supported and have dedicated time and expectations set around the goals of the IPC program, but also that there are enough core personnel in the program (e.g., IPs, associate physicians, data analysts) to ensure the full expected scope of activities for an effective program are met. In instances where such facilities are part of a larger system, synergy of these roles and goals and sharing and standardization of tools and policies may help streamline the work of the various facility IPC teams.
	**Ambulatory surgical center**	Similar to the smaller acute care facility, the resources and staffing necessary to attain an effective program may still be small (e.g., one IP). The IP should have enough time to serve as the strategic dyad IP Director of the program as well as conduct the day-to-day IP activities. If the IP has added facility expectations (e.g., serves as the occupational health personnel), the facility must ensure that the single individual can meet the expectations and conduct all of the activities necessary to be effective in both roles. In addition, a physician partner for the program should be identified, such as a lead surgeon. In this role, these individuals should have expectations for added training in IPC and dedicated time and expectations devoted to IPC program in an active and engaged manner.

A key strategy to ensuring adequate IPC program resources is establishing a baseline number of trained and dedicated personnel required to perform their basic roles.^94^ The IPC program must have ample dedicated time, resources, and compensation to devote to their roles. Through the use of tools like the APIC Staffing Calculator and the framework noted in Section 2, this baseline can be estimated by using the number of patient beds covered by the program, acuity of patients and types of service lines (eg, bone marrow and solid organ transplant, critical care, neonatology), ambulatory patient volume, and anticipated IPC program scope of work. One challenge with IPC program staffing is that many programs are staffed for their expected daily work but not for the intermittent surges of work that come with identification of outbreaks, emergence of novel or high-consequence pathogens, mechanical or facility breakdowns (eg, water intrusions, construction, humidity, or temperature issues), and regulatory surveys and citations.

There are also varying structures for staffing an ideal IP department. Some centers have larger IP departments with multiple personnel. Others rely heavily on a shared model in which there are a smaller number of core personnel, and staff from clinical units fulfill a liaison role to perform standard IPC services (eg, training, practice audits), or some combination.^95,96^ Use of light duty staff (off on restricted work due to injury), nursing and health sciences and other graduate students, and offering of part-time program roles (eg, individuals who serve as auditors of hand hygiene or environmental cleaning practices) can also be used to offload IPC program members to free them up for other work and allow them to perform at the top of their licenses. A summary of survey responses from a single center in the Netherlands highlighted that Infection Control link nurses were empowered to initiate HAI prevention activities, transfer learned skills between wards, and provide peer feedback to improve IPC policy adoption and compliance.^97–99^ While this model may allow for IPC structured programs in settings with limited resources, it may be less ideal during periods of increased needs at the facility or staffing shortages, such as was incurred during the COVID-19 pandemic. In an ideal program, IPC personnel numbers should be sufficient to maintain baseline IPC activities while anticipating the expected vacations, extended leaves, and other personnel restrictions that will arise.

It is important to recognize that for several years, IPC programs have had staffing shortages and an ageing population nearing retirement.^100^ The impact of the COVID-19 pandemic led to further dissolution of IPC programs or trimming down of personnel. In addition, the percentage of ID fellowship positions filled has declined in the last few years despite a stable number of applicants.^101–104^ Some areas, such as in more rural and underserved communities, may have no access to ID physicians or IP experts.^105^ In 2017, nearly 80% of counties in the U.S. did not have a single ID physician. These factors not only threaten the previously discussed positive impacts of IPC programs but also the call for such programs to be even more active and effective. Efforts aimed at fostering and enhancing the IPC workforce, including improving the pipeline of IPs, recruiting more trainees into ID with associated improvements in compensation, and strengthening the diversity of the IPC workforce are essential long-term strategies.

Considering these shortages, facilities may need tools and strategies to attain such expertise for their IPC programs. Non-ID physicians may need to serve as Medical Director of the IPC program, but it is then essential that such individuals are supported to acquire key training in the previously noted core competencies or have supporting personnel with those competencies in the program. The use of teleconsultative models to share physician or IP expertise are options to provide smaller IPC programs with access to these skilled professionals, although such a structure will reduce the benefit that is attained through having an in-person presence at the facility who can more easily interact and partner with frontline providers and leaders.

Shared personnel pools within a facility may allow for cross-trained staff to function in parallel roles and pivot to dedicated IPC needs as opportunities and unexpected events arise. In addition, champion extenders who are non-IP professionals embedded in clinical units and acting as a direct resource on limited IPC matters have found some success in reducing HAIs. The APIC competency model can serve as a rapid-training tool for these staff members or provide professional development goals for established IPs to support retention.^66^ Identifying former IPs who have transitioned to other roles, retired, or left the organization could serve as a ready reserve of temporary expert help in the event of unexpected issues (eg, disease outbreaks).

Outside of shared staffing models, IPC programs can effectively use multidisciplinary or interprofessional teams to obtain and optimize additional resources. These interprofessional teams may be largely determined by organization size and institutional priorities; however, successful teams have promoted informal interactions, culture of accountability, and interprofessional rounding to optimize HAI prevention efforts.^106^ Additionally, local and state public health professionals may provide vital support in terms of knowledge base and content expertise for IPC programs, especially in rural areas or for long-term acute care facilities.^107^

Technology offers multiple ways to improve and streamline IPC practices, including centralizing HAI detection and surveillance into a single platform, using electronic hand hygiene audit tools, and utilization of AI models or large language processing to educate, train, and test clinical staff.^108^ Additionally, trainings on IPC content for temporary hires, unit staff, trainees, or IPC core team members could be developed as computer-based modules utilizing internet-based tools to assess knowledge,^109^ which can lessen the burdens on IPC teams to train others. For larger health systems, or those outsourcing IP roles, centralized teams can access the IPC electronic platforms and perform remote tasks (eg, HAI surveillance) at multiple sites, allowing a potentially more financially palatable fractional FTE at several sites. This may also free up the on-site IPC personnel to focus on tasks that require and benefit from their physical presence. Maximizing use of electronic tools can streamline work and ultimately improve patient outcomes as well as IPC staff satisfaction.

Larger entities and systems could develop a core IPC strike team of physician and IP experts, easily deployable to facilities in need. This team could provide targeted, prompt, and effective responses to urgent situations. Any intervention that pulls IPC personnel from their normal work, however, would need to include a plan to return them to their normal roles as soon as possible to avoid burnout. Concurrently, leveraging local, regional, and/or national resources that are external to the organization can provide additional support. This can include partnerships with other tele-networks for remote support, pulling in external healthcare providers that may be affiliated with other institutions to staff the IPC program, or seeking accredited private or community organizations that can perform routine IPC activities to maximize available options for IPC programs.^110^ Healthcare facilities that are part of larger health systems may have access to system-level IPC leaders and harmonized tools and policies, which can streamline IPC efforts at each facility. A larger discussion on implementing effective IPC program recommendations within larger health systems will be the focus of a partner paper.

Finally, if an emergent IPC need arises, a sub-optimal but intermittently necessary option may be to temporarily reduce standard IPC work to free up personnel or resources. While this may be a practical approach, it runs counter to our push to move IPC programs to a more resilient and effective framework that addresses all infectious harms. Unless there are exemptions from regulatory authorities, it is imperative that if such a scaling back of IPC activities occurs, there is a clearly delineated path to restarting routine IPC activities as soon as possible once the emergency has stabilized. A key lesson from managing numerous issues during the COVID-19 pandemic is that institutions conducting pandemic preparedness activities should account for their IPC capacities and develop tiered approaches to managing emergencies, addressing insufficient supplies, training redeployed staff, and maximizing communication.^94,111,112^ As has been done with mandatory participation in hand hygiene metrics or *Staphylococcus aureus* bacteremia prevention, national and regional regulatory agencies may assist in IPC optimization by requiring institutions to report preparedness plans emphasizing steps to optimize scarce IPC resources.^113^

These strategies highlight several ways to optimize limited resources in IPC programs, each offering unique benefits and considerations. Implementing these strategies requires careful planning and coordination and may enhance the effectiveness and efficiency of IPC programs. Implementing these strategies toward the goal of developing a maximally effective IPC program can improve patient outcomes, lead to cost avoidance, and positively impact important financial, operational, and reputational metrics. It must be emphasized that the most effective IPC programs are staffed appropriately at baseline, including staffing for unexpected issues and emergencies, rather than depending on extraordinary measures simply because they are understaffed and under resourced.

## Conclusions

Great strides have been made in the past 50 years toward reducing infectious harms, including HAIs, in healthcare facilities. Resources, incentives, and accountability for improved infection prevention efforts have been instrumental in improving the quality of care and the safety of patients, HCP, and visitors. Nonetheless, many IPC programs in the U.S. have many more opportunities to reduce harms and positively improve many core outcomes for their patients and facilities. **We call on all interested parties to strengthen the expectations, resources, and commitment to IPC programs to further improve the health, safety, and quality of care as well as critical operations of the facility.** These groups include healthcare facility leaders who support and resource IPC programs and regulatory surveyors and quality evaluators who evaluate, rate, and cite facilities. Requiring and resourcing IPC programs with expertise in leadership roles, a dyad leadership structure, support from accountable partners within the organization, and core members staffed and provided adequate dedicated time to fully address the populations at risk raises the bar of expectations for IPC programs, moving them from “active” to active and fully effective work. Future steps following this position paper will include tools and training for best practices to assist IPC programs, healthcare facility leaders, and other core partners in applying these recommendations to their facilities.

## Supporting information

Talbot et al. supplementary material Talbot et al. supplementary material

## Supplementary material

The supplementary material for this article can be found at https://doi.org/10.1017/ice.2025.73

## References

[1] Magill SS , O’Leary E , Janelle SJ , et al. Changes in prevalence of health care-associated infections in U.S. hospitals. N Engl J Med 2018;379:1732–1744. 30380384 10.1056/NEJMoa1801550 PMC7978499

[2] Gidey K , Gidey MT , Hailu BY , Gebreamlak ZB , Niriayo YL. Clinical and economic burden of healthcare-associated infections: A prospective cohort study. PLoS One 2023;18:e0282141. 36821590 10.1371/journal.pone.0282141 PMC9949640

[3] Roberts RR , Scott RD 2nd, Hota B , et al. Costs attributable to healthcare-acquired infection in hospitalized adults and a comparison of economic methods. Med Care 2010;48:1026–1035. 20940650 10.1097/MLR.0b013e3181ef60a2

[4] Forrester JD , Maggio PM , Tennakoon L. Cost of Health Care-Associated Infections in the United States. J Patient Saf 2022;18:e477–e479. 33881808 10.1097/PTS.0000000000000845

[5] CDC, NHSN. Current HAI Progress Report. Updated Nov. 4, 2022. 2022. https://www.cdc.gov/hai/data/portal/progress-report.html

[6] Agency for Healthcare Research and Quality. Estimating the Additional Hospital Inpatient Cost and Mortality Associated With Selected Hospital-Acquired Conditions. Accessed August 28, 2024, https://www.ahrq.gov/hai/pfp/haccost2017-results.html

[7] The Leapfrog Group. Leapfrog Hospital Surveys. https://www.leapfroggroup.org/data-users/leapfrog-hospital-survey

[8] US News and World Report. Surveys. https://www.usnews.com/topics/subjects/surveys

[9] Condition of participation: Infection prevention and control and antibiotic stewardship programs. 42 CFR 482.42 (2019). https://www.ecfr.gov/current/title-42/part-482/section-482.42

[10] CDC, HAIs. https://www.cdc.gov/healthcare-associated-infections/?CDC_AAref_Val=https://www.cdc.gov/hai/data/archive/data-summary-assessing-progress.html

[11] Baker MA , Sands KE , Huang SS , et al. The impact of coronavirus disease 2019 (COVID-19) on healthcare-associated infections. Clin Infect Dis 2022;74:1748–1754. 34370014 10.1093/cid/ciab688 PMC8385925

[12] Lastinger LM , Alvarez CR , Kofman A , et al. Continued increases in the incidence of healthcare-associated infection (HAI) during the second year of the coronavirus disease 2019 (COVID-19) pandemic. Infect Control Hosp Epidemiol 2023;44:997–1001. 35591782 10.1017/ice.2022.116 PMC9237489

[13] Coffey KC , Keller SC , Anderson DJ , et al. Infection prevention and antibiotic stewardship program needs and practices in 2021: a survey of the society for healthcare epidemiology of america research network. Infect Control Hosp Epidemiol 2023;44:948–950. 36916202 10.1017/ice.2022.222 PMC10262154

[14] Rechtman L , Pickrell B. Components Necessary for an Effective Infection Prevention and Control Program McKing Consulting Corporation Corresponding Author. *preprint*. 2024.

[15] SHEA. The Society for Healthcare Epidemiology of America (SHEA) Handbook for SHEA-Sponsored Guidelines and Expert Guidance Documents. Accessed August, 2021. https://shea-online.org/wp-content/uploads/2022/02/2022-Handbook-Update-Approved-Posted.pdf

[16] SHEA. The Society for Healthcare Epidemiology of America (SHEA) Handbook for SHEA-Sponsored Guidelines and Expert Guidance Documents. Accessed January, 2017. http://www.shea-online.org/images/docs/2017_Handbook.pdf

[17] Haley RW , Culver DH , Morgan WM , White JW , Emori TG , Hooton TM. Increased recognition of infectious diseases in US hospitals through increased use of diagnostic tests, 1970-1976. Am J Epidemiol 1985;121:168–181. 4014114 10.1093/oxfordjournals.aje.a113989

[18] The Joint Commission. New and Revised Requirements for Infection Prevention and Control for Critical Access Hospitals and Hospitals *R3 Report: Requirement, Rationale, Reference*. 2023:1-8. October 2, 2024, https://www.jointcommission.org/-/media/tjc/documents/standards/r3-reports/r3_report_ic_rewrite-hap_cah.pdf

[19] Centers for Disease Control and Prevention. State-specific supplement to the National Healthcare-Associated Infection Standardized Infection Ratio Report: July 2009 through December 2009. https://archive.cdc.gov/www_cdc_gov/hai/pdfs/stateplans/state-specific-hai-sir-july-dec2009r.pdf

[20] Centers for Disease Control and Prevention. Monitoring hospital-acquired infections to promote patient safety--United States, 1990-1999. MMWR Morb Mortal Wkly Rep 2000;49:149–153. 10737441

[21] Centers for Disease Control and Prevention. Data Summary of HAIs in the US: Assessing Progress 2006-2016. October 2, 2024. Updated December 5, 2017. 2024. https://archive.cdc.gov/#/details?url=https://www.cdc.gov/hai/data/archive/data-summary-assessing-progress.html

[22] Fabre V , Davis A , Diekema DJ , et al. Principles of diagnostic stewardship: a practical guide from the society for healthcare epidemiology of America diagnostic stewardship task force. Infect Control Hosp Epidemiol. 2023;44:178–185. 36786646 10.1017/ice.2023.5

[23] Solanky D , Juang DK , Johns ST , Drobish IC , Mehta SR , Kumaraswamy M. Using diagnostic stewardship to reduce rates, healthcare expenditures and accurately identify cases of hospital-onset Clostridioides difficile infection. Infect Control Hosp Epidemiol 2021;42:51–56. 32943129 10.1017/ice.2020.375 PMC9215221

[24] Watson KJ , Trautner B , Russo H , et al. Using clinical decision support to improve urine culture diagnostic stewardship, antimicrobial stewardship, and financial cost: a multicenter experience. Infect Control Hosp Epidemiol 2020;41:564–570. 32131910 10.1017/ice.2020.37

[25] Rupp ME , Cavalieri RJ , Marolf C , Lyden E. Reduction in blood culture contamination through use of initial specimen diversion device. Clin Infect Dis 2017;65:201–205. 28379370 10.1093/cid/cix304 PMC5849098

[26] Self WH , Mickanin J , Grijalva CG , et al. Reducing blood culture contamination in community hospital emergency departments: a multicenter evaluation of a quality improvement intervention. Acad Emerg Med 2014;21:274–282. 24628752 10.1111/acem.12337 PMC3984048

[27] Batabyal RA , Zhou JJ , Howell JD , et al. Impact of New York state influenza mandate on influenza-like illness, acute respiratory illness, and confirmed influenza in healthcare personnel. Infect Control Hosp Epidemiol 2017;38:1361–1363. 28826427 10.1017/ice.2017.182

[28] Talbot TR , Babcock H , Caplan AL , et al. Revised SHEA position paper: influenza vaccination of healthcare personnel. Infect Control Hosp Epidemiol 2010;31:987–995. 20807037 10.1086/656558

[29] Kuster SP , Boni J , Kouyos RD , et al. Absenteeism and presenteeism in healthcare workers due to respiratory illness. Infect Control Hosp Epidemiol 2021;42:268–273. 33239124 10.1017/ice.2020.444

[30] Widera E , Chang A , Chen HL. Presenteeism: a public health hazard. J Gen Intern Med 2010;25:1244–1247. 20549378 10.1007/s11606-010-1422-x PMC2947637

[31] Chiu S , Black CL , Yue X , et al. Working with influenza-like illness: Presenteeism among US health care personnel during the 2014-2015 influenza season. Am J Infect Control 2017;45:1254–1258. 28526310 10.1016/j.ajic.2017.04.008 PMC5670002

[32] Dick AW , Perencevich EN , Pogorzelska-Maziarz M , Zwanziger J , Larson EL , Stone PW. A decade of investment in infection prevention: a cost-effectiveness analysis. Am J Infect Control 2015;43:4–9. 25564117 10.1016/j.ajic.2014.07.014 PMC4743241

[33] Arefian H , Vogel M , Kwetkat A , Hartmann M. Economic evaluation of interventions for prevention of hospital acquired infections: a systematic review. PLoS One 2016;11:e0146381. 26731736 10.1371/journal.pone.0146381 PMC4701449

[34] Mena Lora AJ , Ali M , Krill C , Spencer S , Takhsh E , Bleasdale SC. Impact of a hospital-wide huddle on device utilisation and infection rates: a community hospital’s journey to zero. J Infect Prev 2020;21:228–233. 33408760 10.1177/1757177420939239 PMC7745585

[35] Centers for Medicare & Medicaid Services. Hospital-Acquired Condition Reduction Program. https://www.cms.gov/medicare/payment/prospective-payment-systems/acute-inpatient-pps/hospital-acquired-condition-reduction-program-hacrp 10.1001/jama.2015.8609 26219055

[36] Centers for Medicare & Medicaid Services. The Hospital Value-Based Purchasing (VBP) Program. https://www.cms.gov/medicare/quality/value-based-programs/hospital-purchasing

[37] Harper B. Best Children’s Hospitals 2023-2024 Honor Roll and Overview. US News and World Report. https://health.usnews.com/health-news/best-childrens-hospitals/articles/best-childrens-hospitals-honor-roll-and-overview

[38] Aboumatar H , Ristaino P , Davis RO , et al. Infection prevention promotion program based on the PRECEDE model: improving hand hygiene behaviors among healthcare personnel. Infect Control Hosp Epidemiol 2012;33:144–151. 22227983 10.1086/663707

[39] Cronin CE , Singh S , Franz B. The impact of Maryland’s payment reforms on hospital community benefit efforts. Health Care Manage Rev 2022;47:E11–E20. 33507040 10.1097/HMR.0000000000000305

[40] Cronin CE , Singh SR , Turner JS , Evashwick CJ. Hospitals’ contributions to their communities: Should they be regulated? Front Public Health. 2022;10:1064210. 36504974 10.3389/fpubh.2022.1064210 PMC9730019

[41] Tan C , Ofner M , Candon HL , et al. An ethical framework adapted for infection prevention and control. Infect Control Hosp Epidemiol 2023;44:2044–2049. 37424230 10.1017/ice.2023.121 PMC10755160

[42] Pogorzelska-Maziarz M , Monsees E , Hessels A. APIC Megasurvey 2020: Methodology and overview of results. Am J Infect Control 2023;51:241–247. 36535317 10.1016/j.ajic.2022.12.002

[43] Reese SM , Gilmartin HM. Infection prevention workforce: Potential benefits to educational diversity. Am J Infect Control 2017;45:603–606. 28549512 10.1016/j.ajic.2017.03.029

[44] Weber DJ , Sickbert-Bennett EE , DiBiase LM , et al. A new paradigm for infection prevention programs: An integrated approach. Infect Control Hosp Epidemiol 2023;44:144–147. 35831916 10.1017/ice.2022.94

[45] Clouser JM , Vundi NL , Cowley AM , et al. Evaluating the clinical dyad leadership model: a narrative review. J Health Organ Manag 2020. 10.1108/JHOM-06-2020-0212 32888264

[46] Saxena A. Challenges and success strategies for dyad leadership model in healthcare. Healthc Manage Forum 2021;34:137–148. 33016128 10.1177/0840470420961522

[47] Zismer DK , Brueggemann J. Examining the “dyad” as a management model in integrated health systems. Physician Exec 2010;36:14–19. 20175382

[48] Leach L , Hastings B , Schwarz G , et al. Distributed leadership in healthcare: leadership dyads and the promise of improved hospital outcomes. Leadersh Health Serv (Bradf Engl) 2021. 10.1108/LHS-03-2021-0011 34245498

[49] Kuntz L , Scholten N , Wilhelm H , Wittland M , Hillen HA. The benefits of agreeing on what matters most: Team cooperative norms mediate the effect of co-leaders’ shared goals on safety climate in neonatal intensive care units. Health Care Manage Rev 2020;45:217–227. 30418291 10.1097/HMR.0000000000000220

[50] Hemker RA , Solomon LA. Building a physician culture for healthcare transformation: a hospital’s leadership challenge. Front Health Serv Manage. Spring 2016;32:3–14. 27125045

[51] Clausen C , Lavoie-Tremblay M , Purden M , Lamothe L , Ezer H , McVey L. Intentional partnering: a grounded theory study on developing effective partnerships among nurse and physician managers as they co-lead in an evolving healthcare system. J Adv Nurs 2017;73:2156–2166. 28251675 10.1111/jan.13290

[52] Baldwin KS , Dimunation N , Alexander J. Health care leadership and the dyad model. Physician Exec 2011;37:66–70. 21827104

[53] Kuntz L , Scholten N , Wilhelm H , Wittl, M , Hillen HA. The benefits of agreeing on what matters most: Team cooperative norms mediate the effect of co-leaders’ shared goals on safety climate in neonatal intensive care units. Health Care Manage Rev. 2020;45:217–227. 30418291 10.1097/HMR.0000000000000220

[54] Robinson J , Price L , Otter J , Burnett E. Designing an optimal infection prevention service: Part 2. J Infect Prev 2023;24:11–22. 36644523 10.1177/17571774221127573 PMC9834426

[55] Halton K , Hall L , Gardner A , MacBeth D , Mitchell BG. Exploring the context for effective clinical governance in infection control. Am J Infect Control 2017;45:278–283. 27916342 10.1016/j.ajic.2016.10.022

[56] Goodall AH. Physician-leaders and hospital performance: is there an association? Soc Sci Med. 2011;73:535–539. 21802184 10.1016/j.socscimed.2011.06.025

[57] Wright SB , Ostrowsky B , Fishman N , Deloney VM , Mermel L , Perl TM. Expanding roles of healthcare epidemiology and infection control in spite of limited resources and compensation. Infect Control Hosp Epidemiol 2010;31:127–132. 20039800 10.1086/650199

[58] Dhar S , Sandhu AL , Valyko A , Kaye KS , Washer L. Strategies for effective infection prevention programs: structures, processes, and funding. Infect Dis Clin North Am 2021;35:531–551. 34362533 10.1016/j.idc.2021.04.001

[59] Kaye KS , Anderson DJ , Cook E , et al. Guidance for infection prevention and healthcare epidemiology programs: healthcare epidemiologist skills and competencies. Infect Control Hosp Epidemiol 2015;36:369–380. 25998192 10.1017/ice.2014.79

[60] The Joint Commission. New and Revised Requirements for Infection Prevention and Control for Critical Access Hospitals and Hospitals. R3 Report. Issue 41. https://www.jointcommission.org/-/media/tjc/documents/standards/r3-reports/r3_report_ic_rewrite-hap_cah.pdf

[61] Saxena A , Meschino D , Hazelton L , et al. Power and physician leadership. BMJ Leader 2019;3:92–98.

[62] Murphy DM , Hanchett M , Olmsted RN , et al. Competency in infection prevention: a conceptual approach to guide current and future practice. Am J Infect Control 2012;40:296–303. 22541852 10.1016/j.ajic.2012.03.002

[63] Association for Professionals in Infection Control and Epidemiology. Infection preventionist (IP) competency model. https://apic.org/professional-practice/infection-preventionist-ip-competency-model/

[64] Billings C , Bernard H , Caffery L , et al. Advancing the profession: an updated future-oriented competency model for professional development in infection prevention and control. Am J Infect Control 2019;47:602–614. 31146830 10.1016/j.ajic.2019.04.003

[65] Davis J , Billings C , Malik C. Revisiting the association for professionals in infection control and epidemiology competency model for the infection preventionist: an evolving conceptual framework. Am J Infect Control 2018;46:921–927. 29861150 10.1016/j.ajic.2018.04.210

[66] Bernard H , Hackbarth D , Olmsted RN , Murphy D. Creation of a competency-based professional development program for infection preventionists guided by the APIC competency model: steps in the process. Am J Infect Control. 2018;46:1202–1210. 29887164 10.1016/j.ajic.2018.04.225

[67] Association for Professionals in Infection Control and Epidemiology. IP Academic Pathway. https://apic.org/ip-academic-pathway/

[68] Certification Board of Infection Control and Epidemiology I. CIC® Initial Exam Eligibility Requirements. https://www.cbic.org/CBIC/Updated-Eligibility-Requirements.pdf

[69] Marx JF , Callery S , Boukidjian R. Value of certification in infection prevention and control. Am J Infect Control 2019;47:1265–1269. 31128984 10.1016/j.ajic.2019.04.169

[70] Hsu YJ , Zhou Z , Nosakhare E , Marsteller JA. Impact of certified infection preventionists in acute care settings: A systematic review. Am J Infect Control 2023;51:334–339. 35764180 10.1016/j.ajic.2022.06.020

[71] Frankel A , Haraden C , Federico F , Lenoci-Edwards J. A Framework for Safe, Reliable, and Effective Care. White Paper. Institute for Healthcare Improvement and Safe & Reliable Healthcare; 2017.

[72] Clifford RJ , Newhart D , Laguio-Vila MR , Gutowski JL , Bronstein MZ , Lesho EP. Infection preventionist staffing levels and rates of 10 types of healthcare-associated infections: A 9-year ambidirectional observation. Infect Control Hosp Epidemiol 2022;43:1641–1646. 35034676 10.1017/ice.2021.507

[73] Pogorzelska-Maziarz M , Gilmartin H , Reese S. Infection prevention staffing and resources in U.S. acute care hospitals: results from the APIC MegaSurvey. Am J Infect Control 2018;46:852–857. 29861151 10.1016/j.ajic.2018.04.202

[74] O’Boyle C , Jackson M , Henly SJ. Staffing requirements for infection control programs in US health care facilities: Delphi project. Am J Infect Control 2002;30:321–333. 12360140 10.1067/mic.2002.127930

[75] Bartles R , Dickson A , Babade O. A systematic approach to quantifying infection prevention staffing and coverage needs. Am J Infect Control 2018;46:487–491. 29307751 10.1016/j.ajic.2017.11.006

[76] Bryant KA , Harris AD , Gould CV , et al. Necessary infrastructure of infection prevention and healthcare epidemiology programs: a review. Infect Control Hosp Epidemiol. 2016;37:371–380. 26832072 10.1017/ice.2015.333 PMC6481289

[77] World Health Organization. WHO Multi-level global survey on minimum requirements for Infection Prevention and Control (IPC) at the national and health care facility levels. https://www.who.int/news-room/articles-detail/who-multi-level-global-survey-on-minimum-requirements-for-ipc-at-the-national-and-health-care-facility-levels

[78] World Health Organization. Guidelines on core components of infection prevention and control programmes at the national and acute health care facility level. https://www.who.int/publications/i/item/9789241549929 27977095

[79] Association for Professionals in Infection Control and Epidemiology. APIC Staffing Calculator. https://apic.org/apic-staffing-calculator/

[80] Mann T , Ellsworth J , Huda N , et al. Building and validating a computerized algorithm for surveillance of ventilator-associated events. Infect Control Hosp Epidemiol 2015;36:999–1003. 26072660 10.1017/ice.2015.127

[81] Hota B , Jones R , Schwartz D. Informatics and infectious diseases: What is the connection and efficacy of information technology tools for therapy and health care epidemiology? Am J Infect Control. 2008;36:S47–S56.

[82] US Government Accountability Office. VA Health Care: Improved Oversight Needed for Reusable Medical Equipment. https://www.gao.gov/products/gao-18-474

[83] Krause B. Veterans Affairs Fails To Sterilize Surgical Equipment Properly, Again. DisabledVeterans.org. https://www.disabledveterans.org/veterans-affairs-fails-to-sterilize-surgical-equipment-properly-again/

[84] Bouffard K, Kurth J. Dirty, missing instruments plague DMC surgeries. The Detroit News. https://www.detroitnews.com/story/news/special-reports/2016/08/25/dirty-instruments-plague-dmc-surgeries/89303582/

[85] Delmonico K. Instrument sterilization class action settles for $6.5M. This Week in Orthopedics. https://ryortho.com/breaking/instrument-sterilization-class-action-settles-for-6-5m/

[86] The Joint Commission. Top 5 most challenging requirements for 2023. *Joint Commission Online*. April 3, 2024. https://www.jointcommission.org/resources/news-and-multimedia/newsletters/newsletters/joint-commission-online/april-3-2024/top-5-most-challenging-requirements-for-2023/

[87] Collins CD , Dumkow LE , Kufel WD , Nguyen CT , Wagner JL. ASHP/SIDP Joint statement on the pharmacist’s role in antimicrobial stewardship. Am J Health-Syst Pharm 2023;80:1577–1581. 37879095 10.1093/ajhp/zxad164

[88] Diekema DJ , Saubolle MA. Clinical microbiology and infection prevention. Journal of Clinical Microbiology. 2011;49:S57– S60.

[89] Benbachir M. Role of the Microbiology Laboratory in Infection Control. Guide to Infection Control in the Hospital. International Society for Infectious Disease. https://docslib.org/doc/143158/role-of-the-microbiology-laboratory-in-infection-control

[90] Brannigan ET , Murray E , Holmes A. Where does infection control fit into a hospital management structure? J Hosp Infect 2009;73:392–396. 19699008 10.1016/j.jhin.2009.03.031

[91] Bearman G , Stevens MP. Pushing beyond resistors and constipators: implementation considerations for infection prevention best practices. Curr Infect Dis Rep 2014;16:388. 24407546 10.1007/s11908-013-0388-3

[92] Institute of Medicine (IOM). Crossing the Quality Chasm: A New Health System for the 21st Century. National Academy Press; 2001. 25057539

[93] Betsy Lehman Center for Patient Safety and Medical Error Reduction, JSI Research and Training Institute I, Massachusetts Department of Public Health. Prevention and Control of Healthcare-Associated Infections In Massachusetts Part 1: Final Recommendations of the Expert Panel. https://data.patientcarelink.org/uploadDocs/1/Betsy-Leham.pdf

[94] Baswa A , Russo PL , Doyle JS , Ayton D , Stewardson AJ. Experience and perspectives of infection prevention staff of the COVID-19 response in Australian hospitals. Antimicrob Resist Infect Control 2022;11:77. 35655247 10.1186/s13756-022-01116-9 PMC9161183

[95] Thom KA , Li S , Custer M , et al. Successful implementation of a unit-based quality nurse to reduce central line-associated bloodstream infections. Am J Infect Control 2014;42:139–143. 24360354 10.1016/j.ajic.2013.08.006 PMC3946639

[96] Wright J , Stover BH , Wilkerson S , Bratcher D. Expanding the infection control team: development of the infection control liaison position for the neonatal intensive care unit. Am J Infect Control 2002;30:174–178. 11988713 10.1067/mic.2002.119927

[97] Dekker M , Jongerden IP , Caris MG , de Bruijne MC , Vandenbroucke-Grauls C , van Mansfeld R. Evaluation of an infection control link nurse program: an analysis using the RE-AIM framework. BMC Health Serv Res 2023;23:140. 36759832 10.1186/s12913-023-09111-5 PMC9912654

[98] Dekker M , van Mansfeld R , Vandenbroucke-Grauls CM , et al. Role perception of infection control link nurses; a multi-centre qualitative study. J Infect Prev 2022;23:93–100. 35495104 10.1177/17571774211066786 PMC9052847

[99] Dekker M , Jongerden IP , van Mansfeld R , et al. Infection control link nurses in acute care hospitals: a scoping review. Antimicrob Resist Infect Control 2019;8:20. 30705754 10.1186/s13756-019-0476-8 PMC6348687

[100] Rebmann T , Holdsworth JE , Lugo KA , Alvino RT , Gomel A. Impacts of the COVID-19 pandemic on the infection prevention and control field: Findings from focus groups conducted with association for professionals in infection control & epidemiology (APIC) members in fall 2021. Am J Infect Control 2023;51:968–974. 36882121 10.1016/j.ajic.2023.02.013 PMC9985535

[101] Landman K. Great news for germs. The US doesn’t have enough infectious disease doctors — and the situation is about to get worse. VOX News. https://www.vox.com/science-and-health/2022/12/23/23520581/infectious-disease-doctors-physicians-workforce-shortage-match-fellowship-rural-fauci

[102] Ruiz R. Physician and Rep. Raul Ruiz: The infectious disease doctor shortage threatens future pandemic preparedness. STAT. https://www.statnews.com/2023/11/22/infectious-disease-doctor-physicians-residents-pay-raul-ruiz/

[103] Andrews HS , Chirch LM , Luther VP , Shnekendorf R , Nolan NS , Paras ML. Analysis of the infectious diseases fellowship program directors postmatch 2023 survey. J Infect Dis 2024;229:630–634. 38309709 10.1093/infdis/jiad514

[104] Arias CA , Pirofski LA. Infectious diseases training in the 21st century: a glass half full or half empty? J Infect Dis 2024;229:621–624. 38309698 10.1093/infdis/jiad569 PMC10938195

[105] Walensky RP , McQuillen DP , Shahbazi S , Goodson JD. Where Is the ID in COVID-19? Ann Intern Med 2020;173:587–589. 32491920 10.7326/M20-2684 PMC7277486

[106] Gregory ME , MacEwan SR , Sova LN , Gaughan AA , Scheck McAlearney A. A Qualitative examination of interprofessional teamwork for infection prevention: development of a model and solutions. Med Care Res Rev 2023;80:30–42. 35758303 10.1177/10775587221103973 PMC10278586

[107] Wiemken T , Polgreen PM , McKinney WP , Ramirez J , Just E , Carrico R. Knowledge sharing among healthcare infection preventionists: the impact of public health professionals in a rural state. BMC Res Notes 2012;5:387. 22838734 10.1186/1756-0500-5-387 PMC3586955

[108] Wiemken TL , Carrico RM. Assisting the infection preventionist: use of artificial intelligence for health care-associated infection surveillance. Am J Infect Control 2024;52:625–629. 38483430 10.1016/j.ajic.2024.02.007

[109] Desai N , Philpott-Howard J , Wade J , Casewell M. Infection control training: evaluation of a computer-assisted learning package. J Hosp Infect 2000;44:193–199. 10706802 10.1053/jhin.1999.0673

[110] Harrod M , Manojlovich M , Kowalski CP , Saint S , Krein SL. Unique factors rural Veterans’ Affairs hospitals face when implementing health care-associated infection prevention initiatives. J Rural Health 2014;30:17–26. 24383481 10.1111/jrh.12024

[111] Advani SD , Cromer A , Wood B , et al. The impact of coronavirus disease 2019 (COVID-19) response on hospital infection prevention programs and practices in the southeastern United States. Infect Control Hosp Epidemiol 2023;44:338–341. 34725004 10.1017/ice.2021.460 PMC8632447

[112] Greene C , Wilson J , Griffin H , et al. The role of pandemic planning in the management of COVID-19 in England from an infection prevention and control perspective: results of a national survey. Public Health 2023;217:89–94. 36867987 10.1016/j.puhe.2023.01.028 PMC9894767

[113] Grayson ML , Stewardson AJ , Russo PL , et al. Effects of the Australian National Hand Hygiene Initiative after 8 years on infection control practices, health-care worker education, and clinical outcomes: a longitudinal study. Lancet Infect Dis 2018;18:1269–1277. 30274723 10.1016/S1473-3099(18)30491-2

[114] American Hospital Association, Health Forum LLC. Fast Facts on U.S. Hospitals, 2024. Updated January 2024. https://www.aha.org/statistics/fast-facts-us-hospitals

[115] Peterson SM , Harbertson CA , Scheulen JJ , Kelen GD. Trends and characterization of academic emergency department patient visits: a five-year review. Acad Emerg Med 2019;26:410–419. 30102817 10.1111/acem.13550

[116] McDermott K , Liang L. Overview of Operating Room Procedures During Inpatient Stays in U.S. Hospitals, 2018. *Healthcare Cost and Utilization Project (HCUP) Statistical Briefs*. Agency for Healthcare Research and Quality; 2021. https://hcup-us.ahrq.gov/reports/statbriefs/sb281-Operating-Room-Procedures-During-Hospitalization-2018.jsp 34637208

[117] Agency for Healthcare Research and Quality. 2019 National Healthcare Quality and Disparities Report. 2024. https://www.ncbi.nlm.nih.gov/books/NBK579359/

[118] Statista Research Department. U.S. Hospitals - Statistics & Facts. Updated July 18, 2024. Accessed October 2, 2024, https://www.statista.com/topics/1074/hospitals/#statisticChapter

[119] American Trauma Society. Trauma Center Levels Explained. Accessed October 2, 2024, https://www.amtrauma.org/page/traumalevels

[120] Landers T , Davis J , Crist K , Malik C. APIC MegaSurvey: methodology and overview. Am J Infect Control 2017;45:584–588. 28126260 10.1016/j.ajic.2016.12.012

