# SHEA/APIC/IDSA/PIDS multisociety position paper: Raising the bar: necessary resources and structure for effective healthcare facility infection prevention and control programs – CORRIGENDUM

**DOI:** 10.1017/ice.2025.10240

**Published:** 2025-10

**Authors:** Thomas R. Talbot, Christopher Baliga, Rebecca Crapanzano-Sigafoos, Tania N. Bubb, Mohamad Fakih, Thomas G. Fraser, Ibukunoluwa C. Kalu, Vidya Mony, Anupama Neelakanta, Ann-Christine Nyquist, Catherine O’Neal, Jan E. Patterson, David K. Warren, Sharon B. Wright

In this article^1^, there is an error in Table 3. In the row “Trauma facility type,” the trauma levels should be reversed: for Level I, read Level V, and vice versa.
