# Supplementary material for: SHEA/APIC/IDSA/PIDS multisociety position paper: Raising the bar: necessary resources and structure for effective healthcare facility infection prevention and control programs
Source: Infect Control Hosp Epidemiol. 2025 Apr 28;46(7):665–83. doi: 10.1017/ice.2025.73 (PMC12277079; doi:10.1017/ice.2025.73)
Supplement: Talbot et al. supplementary material [file S0899823X2500073Xsup001.docx]

**Supplementary Material**

*SHEA/APIC/IDSA/PIDS Multisociety Position Paper*Raising the Bar: Necessary Resources and Structure for Effective Healthcare Facility Infection Prevention and Control Programs

Contents

[Appendix 1. Review criteria, PICO questions, search strategies, and PRISMA 1](#_Toc191634011)

[Review criteria 1](#_Toc191634012)

[Population, intervention, comparison, outcome (PICO) questions 1](#_Toc191634013)

[Search strategies 2](#_Toc191634014)

[Preferred reporting items for systematic reviews and meta-analyses (PRISMA) 5](#_Toc191634015)

[Appendix 2. Director support reported by SHEA and APIC 6](#_Toc191634016)

[Reported levels of IPC Medical Director support from SHEA, 2021-22^1^ 6](#_Toc191634017)

[Reported Levels of IPC Medical Director Support from APIC, 2021 MegaSurvey^2^ 6](#_Toc191634018)

[References 7](#_Toc191634019)

# Appendix 1. Review criteria, PICO questions, search strategies, and PRISMA

## Review criteria

| Search criteria | January 1, 2000 to March 14, 2023 (date of search), English, abstract required |
| --- | --- |
| Exclusion | Commentary, editorial, letter, trial registry record, clinical trial protocol, antimicrobial stewardship programs, low-middle income countries/low-resource countries, “management” referring to a condition or infection |
| Inclusion | IPC programs and teams in hospital settings, leadership, dyad, co-leadership, training, education, hospital epidemiology |

## Population, intervention, comparison, outcome (PICO) questions

| **Theme** | **PICO Question** | **Population** | **Intervention** | **Comparison** | **Outcome** |
| --- | --- | --- | --- | --- | --- |
| Infection prevention and control (IPC) program^1^ and patient safety | 1. How does the presence of an IPC program in a healthcare facility affect patient safety (relative to proportion of high-risk patient procedures and cases), as measured by: patient length of stay, patient mortality, rates of healthcare-associated infections (HAIs, device-related infections or infections caused by MDROs), and cost per patient? | Patients in healthcare facilities | Existence of IPC program with dedicated staff^1^ | No IPC program | (relative to presence of high-risk patient procedures and cases) patients’ length of stay, mortality, acquisition of HAIs, cost of care |
| IPC programs and occupational safety | 1. How does the presence of an IPC program affect healthcare personnel safety as measured by: needlestick/sharp injury, contagious disease exposures, employee sick days, infection-related worker harms? | HCP working, consulting for, volunteering in healthcare facilities | Existence of IPC program with dedicated staff | No IPC program | Needlestick or sharp injury, contagious disease exposures, employee sick days, infection-related worker harms |
| IPC program resources, day-to-day | 1. What is the minimum resourcing (staff FTE, staff expertise, data and information collection, surveillance, liaising, communication, training frontline HCP, implementation) needed for an IPC program to fulfill its responsibilities on a day-to-day basis? | Patients and HCP in healthcare facilities | Existence of IPC program with dedicated staff | No IPC program or under-resourced IPC program | HAI rates, HCP turnover |
| IPC program resources, during unexpected stressors or crises | 1. What is the minimum resourcing (staff FTE, staff expertise, data and information collection, surveillance, liaising, communication, training frontline HCP, implementation) needed for an IPC program to deal with times of prolonged stress or during emergence of unexpected stressors, both infectious (e.g., novel pathogen preparedness (Ebola, pandemic influenza), COVID-19, measles outbreak, high numbers of seasonal influenza or respiratory pathogen infections, facility outbreak) or non-infectious crisis (e.g., flooding, power supply, hospital equipment supply chain breakdown)? | Patients and HCP in healthcare facilities | Existence of IPC program with dedicated staff^1^ | No IPC program or under-resourced IPC program | Patients’ length of stay, mortality, acquisition of HAIs, cost of care, HCP turnover |
| Healthcare leadership models | 1. How do co-led (“dyad model,” e.g., nurse, physician; pharmacist, physician) unit/program leadership models in healthcare facilities vs. other models affect the performance of a program or unit?   *NOTE*: this may not be specific to IPC programs but can relate to other clinical or quality programs in healthcare. | Patients and HCP in healthcare facilities | Co-led/dyad leadership | Other leadership models | Patient outcomes, HCP retention |
| Leadership training | 1. How does leadership who have training specific to the activities of unit/program (e.g., IPC and healthcare epidemiology training for IPC programs) vs. no specific training affect patient outcomes? | Patients in healthcare facilities | Trained | Not trained | Patient outcomes in that unit/program |

## Search strategies

| **PICO** | **Theme** | **Database** | **Search strategies** |
| --- | --- | --- | --- |
| 1-4 | Infection prevention and control programs and patient safety, occupational safety, resource requirements (day-to-day and during crises) | Medline (via PubMed) | ((("Infection Control"[MeSH Terms] OR "cross infection/prevention and control"[MeSH Terms] OR ("Infection Control"[Title/Abstract:~3] OR "infection prevention"[Title/Abstract:~3] OR "infection management"[Title/Abstract:~3])) AND ("program*"[Title/Abstract] OR "team*"[Title/Abstract]) AND ("Hospitals"[MeSH Terms] OR "Hospital Units"[MeSH Terms] OR ("hospital*"[Title/Abstract] OR "ward*"[Title/Abstract] OR "unit*"[Title/Abstract] OR "hospital-wide"[Title/Abstract]))) NOT "antibiotic stewardship"[Title/Abstract] OR "antimicrobial stewardship"[Title/Abstract] OR "antimicrobial resistance"[Title/Abstract] OR "antibiotic resistance"[Title/Abstract] OR "Comment"[Publication Type] OR "Editorial"[Publication Type] OR "Letter"[Publication Type] |
| 1-4 | Infection prevention and control programs and patient safety, occupational safety, resource requirements (day-to-day and during crises) | Cochrane Library | #1: MeSH descriptor: [Infection Control] explode all trees, 1635  #2: MeSH descriptor: [Cross Infection] explode all trees and with qualifier(s): [prevention & control - PC], 984  #3: (infection NEAR/3 (control OR prevention OR management)):ti,ab,kw, 11125  #4: 11927  #5: (program* OR team*):ti,ab,kw, 173899  #6, #4 AND #5: 1185  #7: MeSH descriptor: [Hospitals] explode all trees, 4874  #8: MeSH descriptor: [Hospital Units] explode all trees, 5842  #9: (hospital* OR ward* OR unit* OR "hospital-wide"):ti,ab,kw, 334005  #10: 334569  #11, #6 AND #10: 566  #12: (“antibiotic stewardship” OR “antimicrobial stewardship” OR “antimicrobial resistance” OR “antibiotic resistance”):ti,ab,kw, 3173  #13: (afghan* OR africa* OR albania* OR algeria* OR angola* OR argentin* OR armenia* OR azerbaijan* OR bangladesh* OR bengal* OR bangal* OR belarus* OR belorus* OR byelarus* OR byelorus* OR belize* OR benin* OR dahomey OR bhutan* OR bolivia* OR bosnia* OR herzegovin* OR botswan* OR batswan* OR bechuanaland* OR brazil* OR brasil* OR bulgaria* OR burkina* OR burundi* OR urundi* OR cambodia* OR kampuchea* OR khmer* OR cameroon* OR cameroun* OR ubangi shari* OR chad* OR china OR chinese OR colombia* OR comoro* OR comore* OR comorian* OR mayotte* OR congo* OR zaire* OR “costa rica” OR cuba OR cuban OR cubans OR djibouti* OR somaliland* OR dominica* OR ecuador* OR egypt* OR “united arab republic” OR “el salvador” OR salvadoran* OR guinea* OR equatoguinea* OR eritrea* OR eswatini* OR swaziland* OR swazi* OR swati* OR ethiopia* OR fiji* OR gabon* OR gabonese* OR gabonaise* OR gambia* OR grenada OR grenadian* OR guatemala* OR guyana* OR guiana* OR guyanese* OR haiti* OR hispaniola* OR hondura* OR india* OR indonesia* OR iran* OR iraq* OR jamaica* OR jordan* OR kazakh* OR kenya* OR karabati* OR korea* OR kosovo* OR kosova* OR kyrgyz* OR kirgiz* OR kirghiz* OR laos OR lao OR laotian* OR lebanon* OR lebanese* OR lesotho* OR basutoland* OR mosotho* OR basotho* OR liberia* OR libya* OR jamahiriya* OR macedonia* OR madagasca* OR malagasy* OR malawi* OR nyasaland* OR malaysia* OR maldives* OR maldivian* OR mali OR malian* OR micronesia* OR marshallese* OR kiribati* OR tuvalu* OR mauritania* OR mauritan* OR mauritius* OR mexico* OR mexican* OR moldova* OR moldovia* OR mongol* OR montenegr* OR morocco* OR moroccan* OR ifni OR mozambique* OR mozambican* OR myanmar* OR burma* OR burmese OR namibia* OR nepal* OR nicaragua* OR niger* OR pakistan* OR palestin* OR gaza* OR panama* OR paraguay* OR peru OR peruvian* OR philippine* OR philipine* OR phillipine* OR phillippine* OR filipino* OR filipina* OR romania* OR russia* OR ussr* OR soviet* OR rwanda* OR rwandese OR ruanda* OR ruandese OR samoa* OR “pacific island” OR “pacific islander” OR polynesia* OR senegal* OR serbia* OR “sierra leone” OR melanesia* OR somali* OR “sri lanka” OR ceylon* OR grenadine* OR sudan* OR surinam* OR syria* OR tajik* OR tadjik* OR tadzhik* OR tanzania* OR tanganyika* OR thai* OR timor OR timorese* OR togo OR togoles* OR tonga* OR tunisia* OR turkiy* OR turkey* OR turk OR turks OR turkish OR turkmen* OR uganda* OR ukrain* OR uzbek* OR vanuatu* OR venezuela* OR vietnam* OR yemen* OR yugoslav* OR zambia* OR zimbabwe* OR rhodesia* OR “middle eastern” OR sahara* OR subsahara* OR magreb* OR maghrib* OR caribbean OR “central America” OR “latin America” OR “south America” OR “South American” OR “Central Asia” OR “Central Asian” OR “eastern Europe” OR “Eastern European” OR lmic OR lmics):ti,ab,kw, 162342  #14, #12 OR #13: 164962  #15, #11 NOT #14: 399 |
| 5 | Co-led healthcare leadership model | Medline (via PubMed) | ((("Leadership"[MeSH Terms] OR ("Delivery of Health Care"[MeSH Terms] AND "Leadership"[Title/Abstract]) OR "Leadership"[Title/Abstract]) AND ("dyad*"[Title/Abstract] OR "co-lead"[Title/Abstract] OR "co-leadership"[Title/Abstract] OR "co-leaders"[Title/Abstract] OR "co-leader"[Title/Abstract] OR "co-led"[Title/Abstract])) NOT ("Comment"[Publication Type] OR "Editorial"[Publication Type] OR "Letter"[Publication Type])) |
| 5 | Co-led healthcare leadership model | Cochrane Library | #1: MeSH descriptor: [Leadership] explode all trees, 391  #2: (leadership):ti,ab,kw, 2426  #3 #1 OR #2: 2426  #4: (dyad* OR "co-leadership" OR "co-leaders" OR "co-leader" OR "co-lead" OR "co-led"):ti,ab,kw, 4244  #5 #3 AND #4 |
| 6 | Infection control and prevention leadership training | Medline (via PubMed) | ((("Infection Control"[MeSH Terms] OR "cross infection/prevention and control"[MeSH Terms] OR "infection control practitioners/education"[MeSH Terms] OR "infection control practitioners/standards"[MeSH Terms] OR "Infection Control"[Title/Abstract:~3] OR "infection prevention"[Title/Abstract:~3] OR "infection surveillance"[Title/Abstract:~3] OR "infection management"[Title/Abstract:~3] OR "healthcare epidemiology"[Title/Abstract] OR "hospital epidemiology"[Title/Abstract] OR "infection epidemiology"[Title/Abstract:~3]) AND ("Education"[MeSH Terms] OR "Professional Competence"[MeSH Terms] OR "Certification"[MeSH Terms] OR ("Education"[Title/Abstract] OR "training"[Title/Abstract] OR "trained"[Title/Abstract] OR "background"[Title/Abstract] OR "Certification"[Title/Abstract] OR "certified"[Title/Abstract])) AND ("leadership"[MeSH Terms] OR ("leader*"[Title/Abstract] OR "preventionist*"[Title/Abstract] OR "manager*"[Title/Abstract] OR "administrator*"[Title/Abstract] OR "director*"[Title/Abstract]))) NOT ("patient education"[Title/Abstract] OR "Patient Education as Topic"[MeSH Terms] OR "Guideline Adherence"[MeSH Terms] OR ("antibiotic stewardship"[Title/Abstract] OR "antimicrobial stewardship"[Title/Abstract] OR "antimicrobial resistance"[Title/Abstract] OR "antibiotic resistance"[Title/Abstract] OR ("Comment"[Publication Type] OR "Editorial"[Publication Type] OR "Letter"[Publication Type]) |
| 6 | Infection control and prevention leadership training | Cochrane Library | #1: MeSH descriptor: [Infection Control] explode all trees  #2: MeSH descriptor: [Cross Infection] explode all trees and with qualifier(s): [prevention & control - PC]#3: MeSH descriptor: [Infection Control Practitioners] explode all trees and with qualifier(s): [standards - ST, education - ED]  #4: (infection NEAR/3 (control OR prevention OR management OR surveillance)):ti,ab,kw  #5: ("healthcare epidemiology" OR "hospital epidemiology" OR (infection NEAR/3 epidemiology)):ti,ab,kw  #6:  #7: MeSH descriptor: [Education] explode all trees  #8: MeSH descriptor: [Professional Competence] explode all trees  #9: MeSH descriptor: [Certification] explode all trees  #10: (education OR training OR trained OR background OR certification OR certified):ti,ab,kw  #11:  #12: MeSH descriptor: [Leadership] explode all trees  #13: (leader* OR preventionist* OR manager* OR administrator* OR director*):ti,ab,kw  #14: #12 OR #13  #15, #6 AND #11 AND #14  #16: ("antibiotic stewardship" OR "antimicrobial stewardship" OR "antimicrobial resistance" OR "antibiotic resistance"):ti,ab,kw  #17: (afghan* OR africa* OR albania* OR algeria* OR angola* OR argentin* OR armenia* OR azerbaijan* OR bangladesh* OR bengal* OR bangal* OR belarus* OR belorus* OR byelarus* OR byelorus* OR belize* OR benin* OR dahomey OR bhutan* OR bolivia* OR bosnia* OR herzegovin* OR botswan* OR batswan* OR bechuanaland* OR brazil* OR brasil* OR bulgaria* OR burkina* OR burundi* OR urundi* OR cambodia* OR kampuchea* OR khmer* OR cameroon* OR cameroun* OR ubangi shari* OR chad* OR china OR chinese OR colombia* OR comoro* OR comore* OR comorian* OR mayotte* OR congo* OR zaire* OR “costa rica” OR cuba OR cuban OR cubans OR djibouti* OR somaliland* OR dominica* OR ecuador* OR egypt* OR “united arab republic” OR “el salvador” OR salvadoran* OR guinea* OR equatoguinea* OR eritrea* OR eswatini* OR swaziland* OR swazi* OR swati* OR ethiopia* OR fiji* OR gabon* OR gabonese* OR gabonaise* OR gambia* OR grenada OR grenadian* OR guatemala* OR guyana* OR guiana* OR guyanese* OR haiti* OR hispaniola* OR hondura* OR india* OR indonesia* OR iran* OR iraq* OR jamaica* OR jordan* OR kazakh* OR kenya* OR karabati* OR korea* OR kosovo* OR kosova* OR kyrgyz* OR kirgiz* OR kirghiz* OR laos OR lao OR laotian* OR lebanon* OR lebanese* OR lesotho* OR basutoland* OR mosotho* OR basotho* OR liberia* OR libya* OR jamahiriya* OR macedonia* OR madagasca* OR malagasy* OR malawi* OR nyasaland* OR malaysia* OR maldives* OR maldivian* OR mali OR malian* OR micronesia* OR marshallese* OR kiribati* OR tuvalu* OR mauritania* OR mauritan* OR mauritius* OR mexico* OR mexican* OR moldova* OR moldovia* OR mongol* OR montenegr* OR morocco* OR moroccan* OR ifni OR mozambique* OR mozambican* OR myanmar* OR burma* OR burmese OR namibia* OR nepal* OR nicaragua* OR niger* OR pakistan* OR palestin* OR gaza* OR panama* OR paraguay* OR peru OR peruvian* OR philippine* OR philipine* OR phillipine* OR phillippine* OR filipino* OR filipina* OR romania* OR russia* OR ussr* OR soviet* OR rwanda* OR rwandese OR ruanda* OR ruandese OR samoa* OR “pacific island” OR “pacific islander” OR polynesia* OR senegal* OR serbia* OR “sierra leone” OR melanesia* OR somali* OR “sri lanka” OR ceylon* OR grenadine* OR sudan* OR surinam* OR syria* OR tajik* OR tadjik* OR tadzhik* OR tanzania* OR tanganyika* OR thai* OR timor OR timorese* OR togo OR togoles* OR tonga* OR tunisia* OR turkiy* OR turkey* OR turk OR turks OR turkish OR turkmen* OR uganda* OR ukrain* OR uzbek* OR vanuatu* OR venezuela* OR vietnam* OR yemen* OR yugoslav* OR zambia* OR zimbabwe* OR rhodesia* OR “middle eastern” OR sahara* OR subsahara* OR magreb* OR maghrib* OR caribbean OR “central America” OR “latin America” OR “south America” OR “South American” OR “Central Asia” OR “Central Asian” OR “eastern Europe” OR “Eastern European” OR lmic OR lmics):ti,ab,kw  #18, #16 OR #17  #19, #15 NOT #18 |

## Preferred reporting items for systematic reviews and meta-analyses (PRISMA)

| **January 2000-March 2023** | |
| --- | --- |
| 4,556 records from Medline and Cochrane search strategies, screened by medical librarian | 3,648 records excluded based on PICO criteria (Medline: 3,464, Cochrane: 184) |
| 911 studies imported for expert panel screening | 2 duplicates removed |
| 909 studies screened (2 reviewers per study; conflicts resolved by first and last authors) | 620 studies irrelevant |
| 289 full-text studies assessed for eligibility | 10 studies excluded |
| 279 studies extracted |  |
| **85 citations in final manuscript** | |

# Appendix 2. Director support reported by SHEA and APIC

## Reported levels of IPC Medical Director support from SHEA, 2021-22^1^

Hospital Epidemiologist FTE to Bed Size; N = 37 healthcare facilities

| **Hospital Epi (IPC Medical Director) FTE** | **Median Beds** | **N (unique facilities)** |
| --- | --- | --- |
| 0.0-0.5 | 300 | 9 |
| 0.6-1.0 | 600 | 11 |
| 1.1-1.5 | 800 | 9 |
| 1.6-2.0 | 604 | 4 |
| 2.1-2.5 | 650 | 2 |
| 2.6-3.0 | 960 | 1 |
| 3.1-3.5 | 1,000 | 1 |

## Reported Levels of IPC Medical Director Support from APIC, 2021 MegaSurvey^2^

N = 552 respondents

| **Facility Bed Size Range** | **Average IPC Medical Director FTE** | **N** |
| --- | --- | --- |
| ≤50 beds | 0.89 | 56 |
| 51-300 beds | 0.88 | 225 |
| ≥300 beds | 1.17 | 271 |

# References

1. Coffey KC, Keller SC, Anderson DJ, et al. Infection prevention and antibiotic stewardship program needs and practices in 2021: A survey of the Society for Healthcare Epidemiology of America Research Network. *Infect Control Hosp Epidemiol*. Jun 2023;44:948-950.

2. Landers T, Davis J, Crist K, Malik C. APIC MegaSurvey: Methodology and overview. *Am J Infect Control*. 2017;45:584-588.
